# Approaches and applications in transdermal and transpulmonary gene drug delivery

**DOI:** 10.3389/fbioe.2024.1519557

**Published:** 2025-01-15

**Authors:** Anni Zhang, Xuran Zhang, Jiahui Chen, Xianbao Shi, Xijuan Yu, Zhonggui He, Jin Sun, Mengchi Sun, Zhijun Liu

**Affiliations:** ^1^ Department of Ultrasound, Shengjing Hospital, China Medical University, Shenyang, Liaoning, China; ^2^ Department of Orthopedics, Fuxin Center Hospital, Fuxin, Liaoning, China; ^3^ School of Pharmacy, Shenyang Pharmaceutical University, Shenyang, Liaoning, China; ^4^ Department of Pharmacy, The First Affiliated Hospital of Jinzhou Medical University, Jinzhou, Liaoning, China; ^5^ Wuya College of Innovation, Shenyang Pharmaceutical University, Shenyang, Liaoning, China; ^6^ Joint International Research Laboratory of Intelligent Drug Delivery Systems, Ministry of Education, Shenyang, Liaoning, China

**Keywords:** gene therapy, transdermal gene delivery, transpulmonary gene delivery, current delivery carrier, approaches and applications, opportunities and outlooks

## Abstract

Gene therapy has emerged as a pivotal component in the treatment of diverse genetic and acquired human diseases. However, effective gene delivery remains a formidable challenge to overcome. The presence of degrading enzymes, acidic pH conditions, and the gastrointestinal mucus layer pose significant barriers for genetic therapy, necessitating exploration of alternative therapeutic options. In recent years, transdermal and transpulmonary gene delivery modalities offer promising avenues with multiple advantages, such as non-invasion, avoided liver first-pass effect and improved patient compliance. Considering the rapid development of gene therapeutics via transdermal and transpulmonary administration, here we aim to summarize the nearest advances in transdermal and transpulmonary gene drug delivery. In this review, we firstly elaborate on current delivery carrier in gene therapy. We, further, describe approaches and applications for enhancing transdermal and transpulmonary gene delivery encompassing microneedles, chemical enhancers, physical methods for transdermal administration as well as nebulized formulations, dry powder formulations, and pressurized metered dose formulations for efficient transpulmonary delivery. Last but not least, the opportunities and outlooks of gene therapy through both administrated routes are highlighted.

## Graphical Abstract

**Figure:**
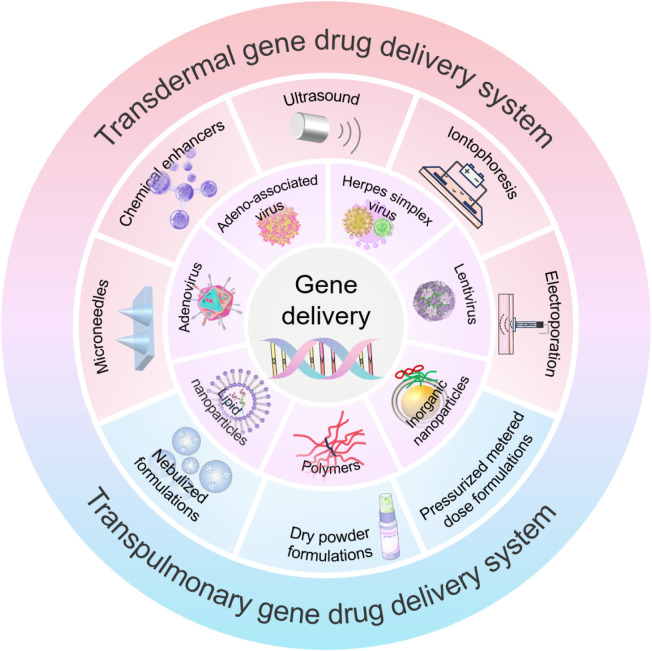
This abstract graphic takes concerted action with abstract section and illustrates the Approaches in transdermal and transpulmonary gene drug delivery.

## 1 Introduction

The concept of gene therapy, was first proposed by Joshua Lederberg in 1963, a therapeutic intervention that modulates the expression of defective proteins by in ducting exogenous genes encoding beneficial proteins into cells to compensate for aberrant genes. Gene therapy did not achieve clinical success until the early 1990s, French Anderson and colleagues utilized *ex vivo* gene therapy for patients with adenosine deaminase deficiency severe combined immunodeficiency (ADA-SCID) by administering injections of T cells transformed by recombinant retroviruses carrying the ADA. This intervention is recognized as the first successful gene therapy in humans. (Authors Anonymous, 1990). Nowadays, the application of gene therapy has emerged as a well-established approach to address diverse human diseases at the genetic level (Wu et al., 2021; Li et al., 2023; Bulcha et al., 2021). The initial scope of gene therapy, which was primarily focused on the treatment of genetic diseases, has now expanded to encompass acquired diseases (Dunbar et al., 2018). However, the successful translation of gene therapy is significantly impeded by various challenges, including the lack of targeted gene delivery to specific disease areas and cells, degradation during the gene delivery process, and rapid clearance within the system (Chen, 2018; Zakrewsky et al., 2015; Pecot et al., 2011). Besides, numerous oligonucleotides currently in clinical development are commonly systemic administered. Nevertheless, injectable patients often exhibit poor compliance and therapeutic efficiency. A diverse range of non-invasive local gene delivery modalities have been developed, including oral, intranasal, pulmonary, cutaneous, ocular, vaginal, and rectal routes (Foldvari et al., 2016). However, the presence of degrading enzymes, acidic pH, and gastrointestinal mucus layer pose challenges for local delivery of genetic material (Chen, 2018). Among them, transdermal and transpulmonary gene delivery employ specific vectors to enhance their penetration through the stratum corneum of the skin and the mucus layer of the airways, offering advantages such as bypassing gastrointestinal influences, enhancing patient compliance and therapeutic potentials (Foldvari et al., 2016; Singh et al., 2022; Prausnitz and Langer, 2008).

In this review, we highlight representative examples of recent materials, formulations, and approaches employed for transdermal and transpulmonary gene delivery. Firstly, we present the primary vectors utilized in gene therapy. Subsequently, we summarize the skin barrier and novel approaches to transdermal drug delivery encompassing chemical enhancers as well as microneedles (MNs) and other modalities. Meanwhile, various inhaled formulations for gene delivery through the lung such as spray formulation and dry powder formulation are elucidated upon. Last but not least, we address the challenges and prospects associated with percutaneous/lung gene delivery.

## 2 Gene therapy

Gene therapy can be categorized into several distinct methods. Gene correction and gene replacement, which leverage gene-editing technologies to identify and excise mutated genes, and cellular DNA repair mechanisms to either correct the mutated genes to normal sequence or facilitate homologous recombination with exogenous, normal genes. Gene inactivation involves the introduction of transcription factors or DNA-binding proteins to suppress the expression of abnormally active genes and thereby treating diseases (Hardee et al., 2017).

Nucleic acids, extensively utilized for gene therapy, encompass DNA and mRNA macromolecules for gene overexpression, as well as smaller entities such as siRNA, miRNA, and antisense oligonucleotides (ASO) for gene knockdown. Gene therapy holds the potential to address diseases that traditional medicine cannot cure. Consequently, it has become a research hotspot in recent years. Although the utilization of naked DNA represents a straightforward approach to gene therapy, its negative charge and the absence of protective vectors increase its vulnerability to nuclease recognition and degradation (Herweijer and Wolff, 2003). Addressing the challenges associated with its susceptibility to nuclease degradation in serum necessitates the implementation of vectors (Santana-Armas and Tros de Ilarduya, 2021). Ideal gene therapy vectors should demonstrate both high reliability and efficiency in delivering therapeutic genes to target cells. This capability is crucial in facilitating long-term expression, ensuring the sustained effectiveness of the treatment (Shirley et al., 2020). Gene therapeutic vectors can be classified into two main groups: viral and nonviral vectors. Each approach comes with its own set of distinct advantages and limitations, which would be explored in the discussion below (Zhang et al., 2021).

### 2.1 Viral vectors

Viral vectors, extensively utilized in gene therapy, are recombinant virus that removed disease-causing genes and maintain the capacity to transfect cells (Santana-Armas and Tros de Ilarduya, 2021; Ibraheem et al., 2014). Until now, a multitude of viral vectors, such as adenovirus (Ad), adeno-associated virus (AAV), and herpes simplex virus (HSV), have demonstrated their potential for secure and effective gene therapy delivery (Zhao et al., 2022). However, viral vectors pose a potential risk of insertional mutagenesis, and may trigger an immune response in the host, potentially limiting the vectors’ reuse and adversely affecting the therapeutic outcome. Owing to the risk of viral transmission, especially in the context of employing replication-competent and soluble tumor viruses, applications involving virus vectors necessitate enhanced levels of biological safety (Lundstrom, 2023). The following section outlines typical viral vectors.

#### 2.1.1 Adenovirus (AdV)

Adenoviruses are non-enveloped double-stranded DNA viruses with an icosahedral structure, measuring 70–100 nm in size (Athanasopoulos et al., 2017). These viruses primarily enter the target cell by binding to various cell surface proteins (Li et al., 2023). In comparison to other viruses, adenoviruses exhibit lower genotoxicity because they do not integrate into the host genome. As a result, they are widely employed as gene therapy vectors (Athanasopoulos et al., 2017).

Recombinant adenoviruses can be rapidly and extensively prepared, showcasing proven genomic stability after successive passages. They exhibit high transduction efficiency and serve as excellent vectors for infectious disease vaccines. Adenoviruses have the ability to infect both quiescent and dividing cells, thereby inducing innate and adaptive immunity. The wide tissue tropism of adenoviruses and their capacity to induce strong expression of target antigens could facilitate their utilization in the development of candidates for infectious diseases (Mendonça et al., 2021). The majority of current vaccines utilize Adenovirus type 5 (Ad5). However, a significant proportion of adults have pre-existing anti-Ad5 antibodies that impede the transduction efficiency of the adenovirus vector.

Adenoviruses are extensively used in gene therapy platforms. Nevertheless, their high immunogenicity poses a challenge to the broad application of adenoviral vectors in treating genetic disorders. In turn, this immunogenicity can be harnessed for cancer immunotherapy applications. This has led to the deployment of adenoviral vectors as vehicles for therapeutic gene transfer, vaccines, and oncolytic agents in the realm of cancer gene therapy (Shaw and Suzuki, 2019). The prevailing trend in adenoviral-based cancer gene therapy focuses on the refinement of adenoviral vectors. For example, adenoviruses can be transformed into oncolytic adenoviruses (OAs). OAs can selectively infect and eradicate cancer cells, resulting in the release of tumor-associated antigens. Thereby recruiting immune cells and subsequently increasing anti-tumor immune responses (Vitale et al., 2022). Various delivery systems, including different cell types and extracellular vesicles, are being investigated for delivering OAs to tumor sites following systemic administration. Moreover, numerous strategies have been devised to enhance the cancer-specific replication ability of OAs, primarily through modifications made to the early region 1 (E1) within the viral genome (Gao et al., 2020).

#### 2.1.2 Adeno-associated virus (AAV)

AAV was initially discovered in laboratory adenovirus (AdV) preparations in the mid-1960s (Atchison et al., 1965). Currently, there are two classes of recombinant AAVs (rAAVs) in use: single-stranded AAV (ssAAV) and self-complementary AAV (scAAV). ssAAVs are packaged as either sense (plus-stranded) or anti-sense (minus-stranded) genomes. These single-stranded forms remain transcriptionally inert upon reaching the nucleus and must undergo conversion to double-stranded DNA before transcription can occur. This conversion can be achieved through second-strand synthesis *via* host cell DNA polymerases or by strand annealing of the plus and minus strands that may coexist in the nucleus. In contrast, scAAVs are already double-stranded by design, allowing them to immediately undergo transcription (Naso et al., 2017).

The capsid protein of AAVs binds to specific receptors on host cells, which confers their natural tropism for different tissues. This inherent preference is crucial for gene therapy, especially for targeting the liver, striated muscles, and the central nervous system (CNS). As a result, the majority of recombinant AAV (rAAV) gene therapy programs focus on these areas to address existing medical challenges, leveraging AAV’s tropism to enhance treatment efficacy and specificity.

Due to the dual blood supply and the highly permeable sinusoid of the liver, nearly all natural AAVs capsids efficiently transduce the hepatocytes upon systemic administration. Consequently, rAAVs serve as a robust platform for liver targeting in the treatment of various diseases, including but not limited to haemophilia A and haemophilia B, familial hypercholesterolaemia, ornithine transcarbamylase deficiency, and Crigler-Najjar syndrome (Ronzitti et al., 2020; Santiago-Ortiz and Schaffer, 2016; Pasi et al., 2020). Capsids like AAV8 and AAV9 demonstrate the ability to target various muscle types throughout the body, thereby facilitating the development of rAAV gene therapies for multiple muscle diseases (Zolotukhin and Vandenberghe, 2022; Verdera et al., 2020), especially those affecting muscles throughout the entire body, such as Duchenne muscular dystrophy (DMD). The rAAV gene therapy strategies include gene replacement, gene silencing, gene addition, and gene editing. Among these vectors, AAVs are the most commonly utilized vectors for delivering the CRISPR/Cas9 system. The rAAV protein capsid, its DNA genome, and the protein product of the transgene can interact with the host immune system at multiple levels, presenting substantial barriers to effective gene delivery and persistent gene expression (Barnes et al., 2019). AAV-based gene therapy faces challenges due to its high cost and the complexity involved in mass production (Naso et al., 2017). The AAV vector, the primary focus of this review, possesses unique features advantageous for clinical applications. These include broad tropism, low immunogenicity, ease of production, non-pathogenicity, rare integration into the host chromosome, and the ability to result in long-term expression of the transgene (Verdera et al., 2020). While AAV vectors have demonstrated initial therapeutic efficacy in clinical settings, concerns have emerged regarding their transduction efficiency and their potential to elicit an immune response against AAV-transduced cells. Vector engineering offers avenues to enhance AAV transduction efficiency through optimization of the transgene cassette, vector tropism through capsid engineering, and the capability of the capsid and transgene to evade the host immune response through genetic modifications of these components. Additionally, vector engineering also aims to optimize the large-scale production of AAV (Pupo et al., 2022).

#### 2.1.3 Herpes simplex virus (HSV)

Herpesviruses have a substantial double-stranded DNA genome ranging from 120 to 230 kilobases. This genome is encased in a protein capsid, enveloped by a tegument layer comprised of viral and host proteins, and surrounded by a lipid bilayer adorned with surface glycoproteins (Mody et al., 2020). Until now, the carrier derived from human herpesvirus has been utilized in vaccines targeting various infectious agents, including the Ebola virus and human immunodeficiency virus (HIV) (Kamel et al., 2023). Herpes simplex virus 1 (HSV-1) is frequently employed as an oncolytic virus (OV) candidate, attributed to its sizable genome, overall safety profile, and capability to infect various cell types (Scanlan et al., 2022). Herpes simplex virus type 1 (HSV-1)-derived amplicon vectors can accommodate large DNA molecules, exhibit minimal toxicity, persist during flares, and pose a negligible risk of insertional mutations. These attributes make them particularly well-suited for gene therapy in neurological diseases due to their capability to deliver genes to neurons and other nerve cells (Fernández-Frías et al., 2020). Achieving sustained therapeutic gene expression in the absence of vector-related toxicity or inflammation is crucial for highly defective HSV vectors. The supplementation of cell lines with carrier production significantly increases the complexity of carrier production (Kuroda et al., 2022). To address this issue, Iván Fernández-Frías et al. have developed methodologies that enhance the production of HSV-1 amplicon, facilitating the preparation of vectors with increased titers and improved purity (Fernández-Frías et al., 2020). HSV-1 amplicon can also serve as a therapeutic carrier driven by a sensory neuron-specific promoter for gene therapy targeting neurogenic detrusor overactivity (NDO) using genetically modified (GM) approaches (Joussain et al., 2022). Despite the immense potential of HSV, certain drawbacks, including limited research and safety concerns, related to the use of herpesvirus vectors in vaccine production. Thurs, a comprehensive understanding of herpesvirus biology is imperative to overcome any persisting limitations (Kamel et al., 2023).

#### 2.1.4 Lentivirus (LV)

**FIGURE 1:**
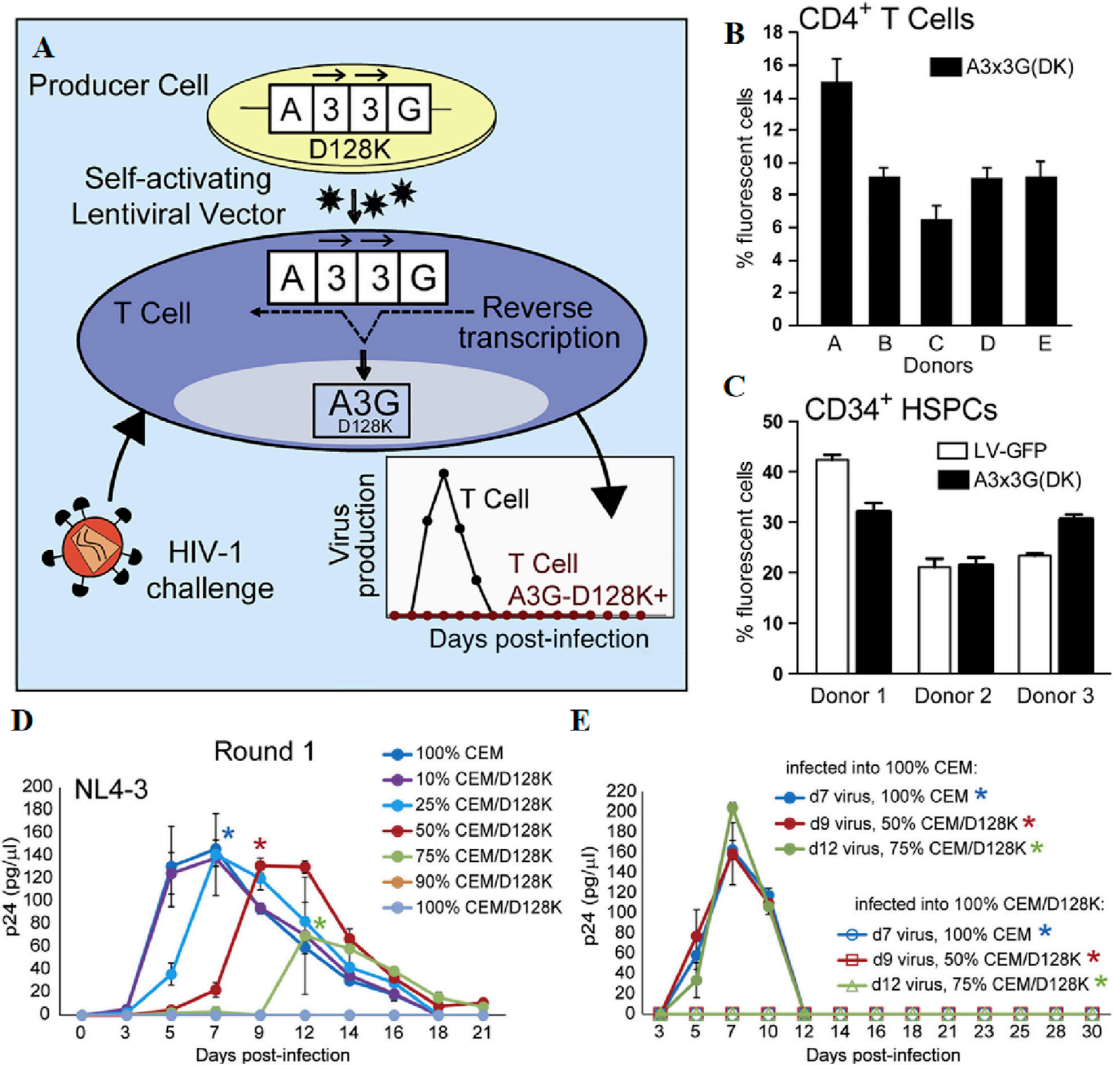
**(A)** Schematic representation of the development of lentiviral vectors for HIV-1 gene therapy. **(B, C)** Efficient Transduction of CD4^+^ T Cells and CD34^+^ HSPCs with the A3x3G-D128K-Expressing Vector. **(D)** NL4-3 virus was infected with different proportions of CEM: CEM/D128K cells and measured by p24 CA ELISA to determine replication kinetics. **(E)** Virus was produced by a first round of cultures, standardized against p24 CA, used to infect 100% CEM or 100% CEM/D128K cells, and the amount of p24 CA was quantified by ELISA to determine replication kinetics. Reprinted with permission from Ref (Delviks-Frankenberry et al., 2019).

LVs, derived from the human immunodeficiency virus, serve as potent tools for the genetic modification of eukaryotic cells, including hematopoietic stem cells and neuronal cells (Wang et al., 2021). LVs are capable of transducing both splinter cells and non-dividing cells, including neurons, hematopoietic stem cells, immune system cells, and T-cells. Notably, they can accommodate genetically modified sequences up to 11,000 bases in size. It is capable of delivering complex therapeutic genes or gene-editing systems, implementing multi-gene therapy strategies, and adapting to various research and treatment requirements (Perry and Rayat, 2021). LVs for gene transfer are now regarded as safer, given their ability to infect non-dividing cells and ensure long-term expression. This characteristic makes them a preferred choice for clinical research (Martínez-Molina et al., 2020). The initial clinical application of lentiviral vectors involved the use of a conditionally replication-competent lentiviral vector encoding an antisense RNA targeting the HIV envelope gene (Milone and O'Doherty, 2018). APOBEC3G (A3G), an intracellular antiviral factor, its function is always inhibited by the Vif protein of HIV-1. Krista A. Delviks-Frankenberry et al. replaced aspartic acid at position 128 with lysine to construct A3G-D128K mutants, which exhibit resistance to the Vif protein. The researchers further developed self-activating LVs to deliver the A3G-D128K to target cells, inducing hypermutations in the HIV genome during reverse transcription and effectively inhibiting viral gene replication (Figure 1) (Delviks-Frankenberry et al., 2019). D.B. Kohn et al. conducted a study on patients diagnosed with clinically severe combined immunodeficiency (ADA-SCID) caused by adenosine deaminase (ADA) deficiency, wherein they employed a gene therapy approach involving *ex vivo* transduction of autologous CD34^+^ hematopoietic stem and progenitor cells (HSPCs) with a self-inactivated lentiviral vector encoding human ADA. The results demonstrated that this *ex vivo* lentiviral HSPC gene therapy exhibited remarkable efficacy, with high overall and event-free survival rates, sustained ADA expression, metabolic correction, and functional immune reconstitution (Kohn et al., 2021).

LVs have the advantage of enabling long-term and stable gene expression while demonstrating minimal production of neutralizing antibodies (Li et al., 2023). LVs, however, exhibit genotoxicity as one of their drawbacks. The issue can be addressed by appropriately modifying LVs to mitigate the risk of insertion-induced mutations (Schenkwein et al., 2020). Furthermore, the genetic modification of T lymphocytes is a crucial aspect in both research and therapy. Traditional lentiviral vectors (LVs) lack selectivity for T cells and are unable to modify quiescent or minimally stimulated cells. To address this limitation, Annika M. Frank et al. introduced a novel CD3-targeting lentiviral vector (CD3-LV) capable of genetically modifying human T lymphocytes without prior activation. This makes it suitable for *in vivo* delivery specifically to T cells (Frank et al., 2020). LVs are also employed in gene therapy for liver hereditary coagulation disorders such as hemophilia. However, LVs exhibit mild acute toxicity and require low drug delivery dosages. Michela Milani et al. utilized the natural inhibitor CD47 to counteract the capture of LVs by professional phagocytes. This approach resulted in the development of phagocytoshielded LVs with enhanced efficiency of hepatocyte gene transfer and reduced activation of acute inflammatory responses (Milani et al., 2019).

### 2.2 Non-viral vectors

**FIGURE 2:**
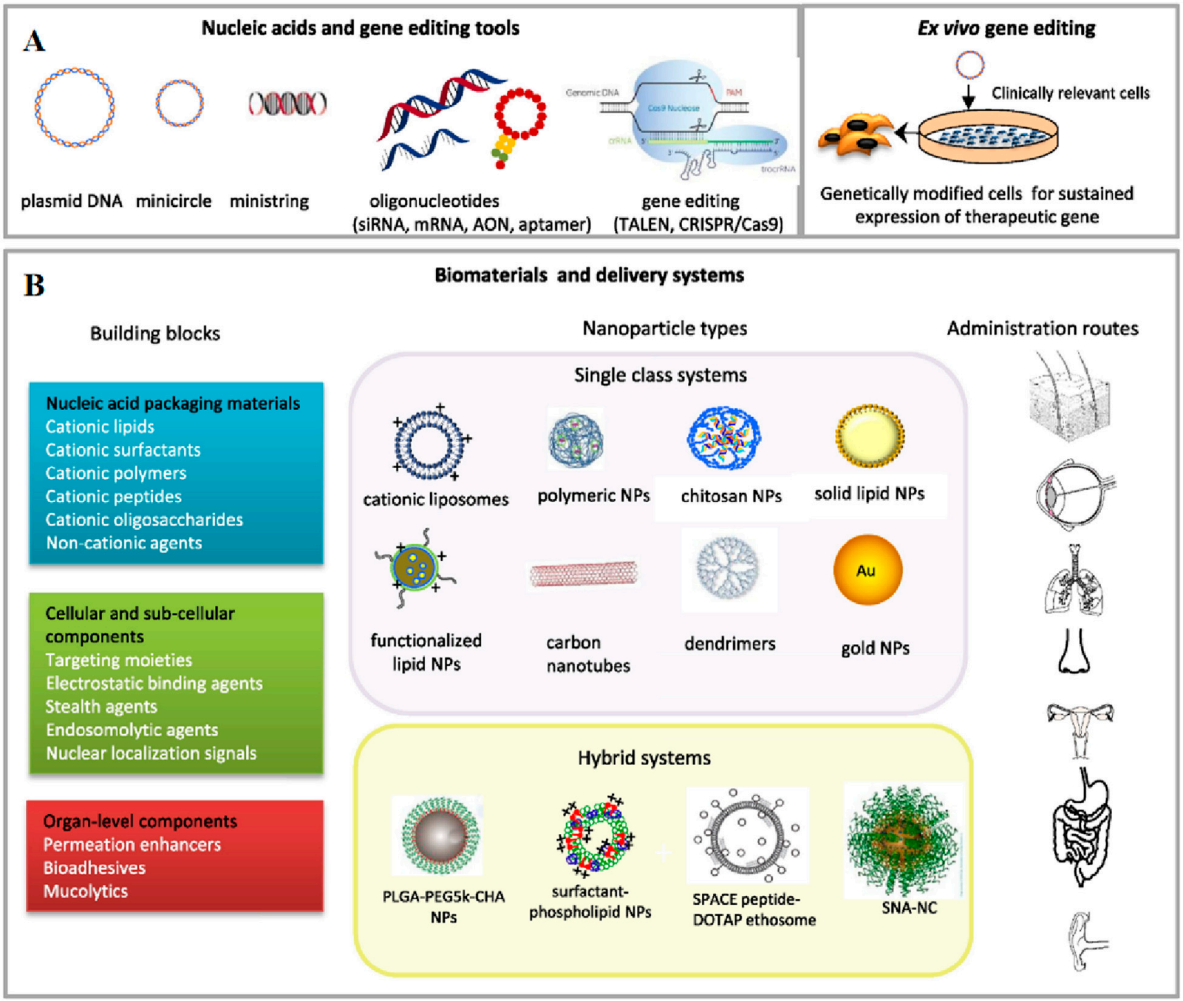
An overview of non-viral gene delivery tools for non-systemic administration. **(A)** Nucleic acids (DNA, therapeutic oligonucleotides and gene editing) and bio- and nanotechnology tools, including *ex vivo* methods, used in non-viral gene therapy. **(B)** Schematic diagram of the building blocks, delivery systems and non-invasive administration routes in gene therapy applications. Reprinted with permission from Ref (Foldvari et al., 2016).

Due to the limited gene delivery capacity of viral vectors and the expensive methods for large-scale production of engineered viruses, non-viral vectors have garnered attention as alternative gene delivery vehicles (Tong et al., 2023; Ren et al., 2021; Mohammadinejad et al., 2020). Non-viral vectors, without viral proteins, possess extremely low immunogenicity and favorable biodegradability mitigates potential cytotoxicity stemming from the prolonged accumulation of the vectors. Though lacking the innate capabilities of virus, non-viral vectors realize cellular uptake by mimicking viral surface properties or triggering membrane fusion by incorporating specific ligands, allowing for the targeted release to enhance the precision of treatment (Ren et al., 2021; Zu and Gao, 2021). Certain vectors are equipped with membrane-active peptides, which can disrupt the endosomal membrane and permit the therapeutic agent to evade into the cytoplasm (Figure 2) (Mohammadinejad et al., 2020). Furthermore, the production and purification for non-viral vectors are typically more straightforward and economical compared to viral vectors, rendering them more cost-effective for large-scale production. The extensively investigated non-viral vectors primarily include lipid nanoparticles, polymers, inorganic nanoparticles and other emerging nanomaterials (Ren et al., 2021; Yan et al., 2022).

#### 2.2.1 Lipid nanoparticles (LNPs)

LNPs serve as prominent non-viral vectors for gene therapy, particularly in mRNA delivery exemplified by the rapid development and approval of mRNA vaccine in COVID-19 pandemic (Wang et al., 2023). Moderna’s mRNA-1273 vaccine utilizes LNPs to deliver mRNA encoding the SARS-CoV-2 spike protein, thereby stimulating an immune response against the virus.

**FIGURE 3:**
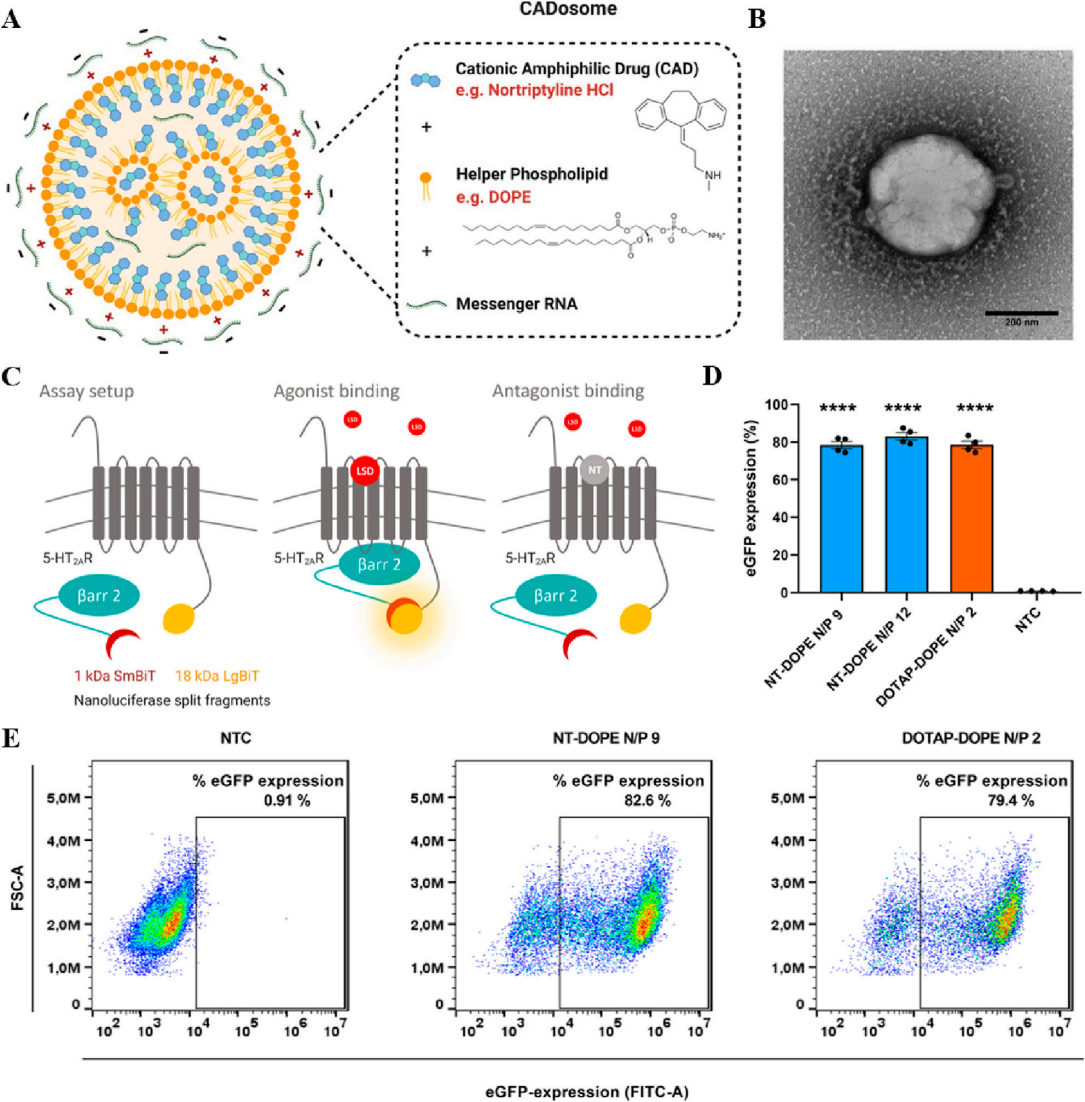
**(A)** Schematic representation of NT-DOPE mRNA CADosomes, produced with vesicles obtained via an ethanol dilution (ED) or lipid film hydration (LFH) method. Created with BioRender.com **(B)** Representative transmission electron microscopy (TEM) image of enhanced green fluorescent protein-encoding messenger RNA (eGFP-mRNA) NT-DOPE CADosomes, prepared via ED. Scale bar corresponds to 200 nm. **(C)** Schematic illustration of the NanoBiT^®^ system. **(D)** Percentage eGFP + cells as analyzed via flow cytometry 24 h after transfection with NT-DOPE CADosomes N/P 9–12 and DOTAP-DOPE N/P 2. **(E)** Representative flow cytometry dot-plots of non-treated cells (NTC) and NT-DOPE CADosomes N/P 9 or DOTAP-DOPE N/P 2 transfected HeLa reporter cells, respectively. Reprinted with permission from Ref (Bogaert et al., 2022).

In gene therapy, endosomes are tasked with transporting the internalized vectors to various cellular compartments, and endosomal escape enables therapeutic nucleic acids to evade the endosome’s acidic milieu, which is essential for subsequent gene expression. LNPs are composed of ionizable lipids, typically uncharged under neutral pH conditions and acquiring a cationic charge within acidic endosomes. This unique property facilitates endosomal escape, thereby ensuring successful mRNA translation. Among the widely employed LNPs, those incorporating cationic or ionizable lipids, cholesterol, auxiliary phospholipids, and pegylated lipids are prevalent (Huang et al., 2022). As versatile nano-delivery vectors, LNPs demonstrate efficacy in delivering cytotoxic chemotherapy drugs, antibiotics, and nucleic acid therapeutics (Jung et al., 2022). Despite this, the intracellular delivery efficiency of state-of-the-art LNPs remains relatively modest, with lingering concerns regarding the safety and immunogenicity of synthetic lipid components. Addressing this challenge, Bram Bogaert et al. presented a novel approach utilizing tricyclic cationic amphiphilic drugs (CADs) as structural and functional components of mRNA-formed lipid NPs. Their study demonstrated that selected CADosomes exhibit highly efficient mRNA delivery in an *in vitro* cell model (Figure 3) (Bogaert et al., 2022). In another breakthrough, Min Qiu et al. engineered a pioneering LNPs delivery platform with specificity, efficacy, and safety for *in vivo* genome editing of Angptl3 by CRISPR-Cas9 (Qiu et al., 2021). *In vivo*, LNPs quickly adsorb plasma proteins to form a protein corona, showing a particular affinity for soluble apolipoprotein E (ApoE). This interaction promotes the binding of LNPs to low-density lipoprotein receptors (LDLr), which are highly expressed on the hepatocytes. Receptor-mediated endocytosis is a key mechanism for the internalization of LNPs by hepatocytes, partially explaining the preferential targeting of the liver by intravenously administered LNPs (Liu et al., 2023). The widespread clinical application of gene therapy has been impeded by the absence of delivery vectors capable of inducing protein expression in extrahepatic organs and tissues (LoPresti et al., 2022). Recent efforts have redirected nucleic acid delivery towards tissues beyond the liver (Kimura and Harashima, 2023). The type and proportion of helper lipids can modify the LNP structure, are essential for the stability and tissue targeting of LNPs. For instance, ester and amide bonds in lipid compounds enhance targeting to the liver and lungs, respectively. Samuel T. LoPresti et al. observed that the complete substitution of conventional helper lipids with anionic or cationic lipids led to significant and consistent transfer of lipid nanoparticles specifically to the spleen or lung, respectively (LoPresti et al., 2022). Beyond their role as delivery components, lipids may exert therapeutic effects synergistically with mRNA-encoded proteins. Multifunctional lipid materials encompass self-adjuvant lipids that enhance vaccine efficacy and paclitaxel-derived lipids enabling the combined treatment of chemotherapy and gene therapy for cancer (Hou et al., 2021; Johnson et al., 2022).

#### 2.2.2 Polymers

Among non-viral vectors, polymer vectors possess the advantages of minimal immunogenicity and easy production, making them optimal alternatives to viral vectors in contemporary research (Chen et al., 2020). The delivery system extensively employs various polymers, including dendritic macromolecules (Luo et al., 2014), polylactic acid (PLA) (Jain et al., 2015), polyethylene imine (PEI) (Peng et al., 2014), and chitosan (CS) (Saeedi et al., 2022; Dong et al., 2024), finding broad applications in the field of cancer therapy and nervous system disease.

The dendritic macromolecules and PLA stand out among gene delivery vehicles due to their non-toxicity, multiple surface functionalities and the capacity to encapsulate and deliver a broad range of therapeutic agents effectively. The high positive charge of PEI promotes endosomal escape of gene complexes and is renowned for its high gene transfection efficiency. CS functions as a natural adhesive polymer, its cationic nature allows for strong interactions with negatively charged cell membranes, which is beneficial for enhancing the residence time and local bioavailability of gene vectors. Pang J et al. prepared DPLL-functionalized amyloss (ADP) and utilized it for the co-delivery of plasmid pIRES2-EGFP-TNFα and curcumin, demonstrating the efficacy of this approach in pancreatic cancer treatment (Pang et al., 2023). Yu-Shiang Peng et al. synthesized PEI-grafted CHI with two different molecular weights (PEI600-g-CHI and PEI1800-g-CHI, Mw = 600 and 1800 g/mol, respectively) through the ring-opening reaction of ethylene glycol diglycidyl ether (EX-810), aiming for Parkinson’s disease (PD) treatment (Peng et al., 2014). Lei Lia et al. developed a cationic polymer, PCL-ssP (PEGMA-co-GMA), as a co-carrier for both anticancer drugs and genes, demonstrating promising prospects for its application (Li et al., 2020). Furthermore, gene delivery can be achieved using composite nanoparticles composed of polymers and cationic peptides. The exceptional transfection efficiency exhibited by Arvind K Jain et al.’s cationic peptide-DNA nanoparticles were synergistically integrated with the biocompatibility and extend release properties inherent in polylactic acid-polyethylene glycol (PLA-PEG). Specifically, the cationic cell-penetrating peptide RALA was utilized to concentrate DNA within nanoparticles, which were subsequently encapsulated in a series of PLA-PEG copolymers. This refined formulation effectively facilitated cellular transfection while preserving cell viability (Jain et al., 2015).

#### 2.2.3 Inorganic nanoparticles

Graham and Bacchetti’s 1983 study, which innovatively employed calcium phosphate as a gene delivery vector, marked a pivotal moment in the field of nonviral gene therapy (Graham and van der Eb, 1973). Nowadays, inorganic nanoparticles (NPs), such as mesoporous silicon nanoparticles (MSNs), gold nanoparticles, magnetic nanoparticles (MNPs), carbon nanotubes, MXenes, and quantum dots (QDs), have emerged as promising vectors for non-viral gene delivery (Patil et al., 2019).

Carbon nanotubes (CNTs), are single-walled or multi-walled tubular nanostructures capable of encapsulating gene molecules in their outer walls and internal cavities, featuring high aspect ratio and high gene-loading capacity (Singh et al., 2005). The tubular nanostructure of CNTs facilitates easier penetration through biological barriers, which is advantageous for the delivery of exogenous plasmids (Talaei and Farzad, 2024). However, the transient expression efficiency of carbon nanotube-mediated exogenous plasmids is undesirable, necessitating further optimization of the delivery system for the carriage and expression of large gene fragments (Hashem Nia et al., 2017).

Silicon-based delivery vectors, such as mesoporous silicon nanoparticles (MSNs), represent a novel type of non-viral gene delivery vectors due to their high loading capacity and stability (Zhou et al., 2018). The mesoporous structure of MSNs provides a large surface area and pore volume, which is advantageous for the accommodation and controlled release of therapeutic genes. Notably, insufficient endosomal escape efficiency of MSNs, necessitating further exploration to enhance the intracellular release of gene fragments (Tong et al., 2019). In addition, the stable framework of MSNs hinders their degradation and excretion, potentially leading to accumulation in vital organs and causing damage.

Magnetic materials, such as Fe3O4, leveraging magnetic targeting under an external field for directional delivery and controlled release of genetic materials (Bi et al., 2020). This strategy holds the promise of dual functionality in both diagnostics and therapeutics (Shasha and Krishnan, 2021). MNPs can be functionalized with various compounds to improve MNPs’ biodistribution and metabolism. Yu et al. utilized PEI--modified MNPs to develop a miRNA-based tumor recognition system, resulting in approximately 42% tumor growth inhibition in mice models (Yu et al., 2016). However, it is important to note that certain MNPs, like CoFe2O4, NiFe2O4, and MnFe2O4, may have limitations due to potential toxicity from metal ion leakage and prolonged exposure to external magnetic fields. These issues require comprehensive research and thoughtful consideration in the application of these systems in biomedicine to ensure safety and efficacy.

Quantum dots (QDs), semiconductor nanocrystals typically range from 2 to 10 nm in diameter. Their photoluminescence properties, ease of functionalization, biocompatibility, and resistance to degradation offer significant advantages for long-term detection and transport within biological organism. However, it is crucial to recognize that metal quantum dots possess inherent cytotoxicity, the mechanisms underlying QDs degradation and clearance remain poorly understood, which could potentially impact the safety and therapeutic efficacy in biomedical applications. Carbon-based quantum dots (CQDs) with their low cytotoxicity and ability to enhance transfection efficiency by decorated with nuclear-targeted peptides, present an exciting alternative in the field of non-viral gene delivery vectors (Zhai et al., 2022).

MXenes are composed of transition metal carbides, carbonitrides, or nitrides. These materials are renowned for their exceptional thinness, large specific surface area, remarkable mechanical strength, and encapsulation capability (Cheng et al., 2020). Concerns persist regarding their stability and biodegradability in physiological environments, potentially leading to long-term accumulation and cytotoxicity. A comprehensive understanding of MXenes’ degradation and clearance mechanisms within the body is an area that merits further research (Bai et al., 2022).

Furthermore, gold nanoparticles exhibit excellent stability, biocompatibility, and facile functionalization. They protect nucleic acids from enzymatic and chemical hydrolysis, thereby enhancing their biological stability to prolong the circulation time (Ren et al., 2021). However, due to their insolubility and propensity for aggregation in biological media, a diverse range of connections and encapsulation is required to render these nanoparticles water-soluble and prevent their aggregation (Xu et al., 2013). In the field of cancer treatment, combination therapy has emerged as a highly effective approach. Nanotechnology-based gene delivery systems offer the potential for co-delivery or simultaneous delivery of multiple therapeutic agents, thereby enhancing existing drug delivery systems through their exceptional drug-loading surface area and passive targeting capability. Binita Shrestha et al. have successfully developed a carrier based on gold nanoparticles for concurrent delivery of drugs and siRNA, which holds great promise for investigating various combinations of drugs and genes (Shrestha et al., 2020). Moreover, gold nanoparticles can also synergize with polymeric chitosan. Xiaoguang Dai et al. developed a generic and straightforward strategy to construct a near-infrared (NIR)-responsive Janus platform for imaging-guided complementary cancer therapy. It is demonstrated that the J-ACP, composed of polycationic chitosan nanospheres and PEGylated gold nanorods, holds significant potential in achieving photoacoustic (PA) imaging-guided complementary photothermal therapy (PTT)/gene therapy for breast cancer (Dai et al., 2021).

## 3 Transdermal gene delivery system

Nucleic acid molecules exhibit sensitivity to various endogenous enzymes within the body, often leading to systemic toxicity and undesirable effects or degradation. To address this predicament, transdermal gene delivery offers a promising approach for achieving high efficacy and low toxicity in genetic medicine (Singh et al., 2022). However, in the implementation of transdermal gene (TG) delivery, the drug must traverse the stratum corneum (SC), which constitutes the outermost layer of the skin, serving as a barrier against the infiltration of foreign molecules into the body (Singh et al., 2022). The skin, recognized as the largest organ in the human body, comprises the epidermis, dermis, and subcutaneous tissue. The linkage between the epidermis and dermis is facilitated by basement membranes anchored by proteins such as collagen, which effectively withstand external shear forces (Zhu et al., 2022). The stratum corneum serves as the primary barrier of the skin (Zhu et al., 2022), selectively allowing penetration of low molecular weight (<500 Da) and highly lipophilic (oil soluble) drugs while effectively preventing transdermal absorption of macromolecules (Yang et al., 2023). In this section, we would present exemplary strategies to surmount these obstacles (Table 1) and discuss their advantages and limitation (Table 2).

**TABLE 1:** Representative examples of transdermal protein delivery systems in different diseases.

Strategies	Approaches	Delivery systems/Delivery strategy	Applications	Evaluation models	Refs
Microneedles	Coated MNs	BRAF siRNA/R8	Melanoma	Mice bearing melanoma	Ruan et al. (2018)
	Dissolving MNs	STAT3 siRNA/PEI	Melanoma	Mice bearing B16F10 melanoma tumor	Pan et al. (2018)
	Degradable MNs	DNA/OSM-(PEG-PAEU)/poly	Cancer	BALB/c female mice	Duong et al. (2018a)
	Metal MNs	Ova/c-di-GMP(c-di-AMP)	Skin disorders	Balb/c female mice	Shakya et al. (2018)
Chemical enhancers	Ionic liquids	siRNA/robed-siRNA	Skin diseases	Female SKH-1E hairless mice	Dharamdasani et al. (2020)
	Biological peptide	DOTAP-based SPACE Ethosomal System	Gene delivery	Female BALB/C mice	Chen et al. (2014)
Physical approaches	Ultrasound	UMGD	Genetic diseases	Specific-pathogenfree (SPF)-derived Yorkshire hybrid swine	Tran et al. (2019)
	Iontophoresis	AuNP-CS/siRNA/CS	melanoma	B16F10 murine melanoma cells	Labala et al. (2016)
	Electroporation	FIEA	Gene delivery	Male C57BL/6 mice	Huang et al. (2050)

**TABLE 2:** Summary of representative transdermal delivery methods.

Strategies	General advantages	General limitation
Microneedles	easy to apply high compliance painless sustained delivery	limited loading capacity suboptimal transfection efficacy
Chemical enhancers	easy to apply low-cost	limited penetration ability potential skin irritation affect nucleic acid activity
Ultrasound	non-invasion painless deep penetration	thermal damage
Electroporation	mature products high penetration ability	high cost complicated operation potential skin damage
Iontophoresis	high penetration ability	high-cost potential skin irritation

### 3.1 Microneedles

Microneedles (MNs) are needle-like structures and an effective approach for gene delivery. It has microscale diameter and lengths up to 1 mm that can penetrate into the stratum corneum (10–40 µm in thickness), and enter the epidermis/dermis layers without touching blood vessels and pain-sensing neurons (Amani et al., 2021). MNs possess several outstanding properties for gene delivery. Firstly, they exhibit minimal invasiveness and can traverse the stratum corneum painlessly. Secondly, MNs can be fabricated and customized in terms of shape, size, and geometry. The material, typically a polymer, can be chosen based on the intended use and release mechanism, showcasing excellent biocompatibility. Dissolvable MNs are crafted from biodegradable polymers such as hydrogel or other dissolving materials, enabling sustained co-delivery of drugs/vaccines and eliminating the necessity for multiple dosing (Singh and Kesharwani, 2021). Thirdly, they enable bypassing first-pass metabolism, facilitating the direct translocation of therapeutics into the systemic circulation (Li et al., 2021). Fourthly, the administration is easy enough to avoid the need for professional training. The development of MNs has resulted in the improvement and expansion of immuno-reprogramming strategies due to the housing of high accumulation of immune cells such as neutrophils (Singh and Kesharwani, 2021), langerhans (Singh and Kesharwani, 2021) dendritic cells, macrophages, lymphocytes, and mast cells in the dermis layer of the skin. These advantages make MNs excellent candidates for the delivery of immunological biomolecules to the dermal antigen-presenting cells in the skin with the aim of vaccinating or treating different diseases, such as cancers and autoimmune disorders, with minimal invasiveness and side effects. Due to the unique properties of the skin, nucleic acid delivery through this tissue holds great potential for treating a wide range of pathologies, including genetic skin conditions, hyperproliferative diseases, cutaneous cancers, wounds, and infections.

DNA vaccines are potentially attractive, but their low immunogenicity has been a barrier to their approval for use. DNA vaccines have demonstrated limited efficacy in clinical trials, owing to the lack of a suitable DNA delivery system. Delivery of DNA vaccines to the highly immune responsive layer of the skin may enhance its immunogenicity. The skin is a significant component of immune system which contains a large amount of antigen presenting cells (APCs). The APCs can transport antigens to lymph nodes and present peptide fragments to lymphocytes which then drive adaptive immune response (Nestle et al., 2009). However, the accuracy and precision of intradermal injection are often low, leading to vaccine preparations frequently leaking out of the skin, thus challenging the induction of an effective immune response. Overall, the delivery of prophylactic vaccines is primarily hindered by factors such as low transfection efficacy, poor immunogenicity, and safety concerns associated with the materials utilized (Duong et al., 2018b). MNs designed for delivering cancer vaccines offer precise delivery of antigens or immune adjuvants to specific skin layers. This targeted approach effectively addresses immune cells in the skin, inducing a more robust immune response compared to intradermal injection, thereby enhancing the therapeutic effect on melanoma. Grace Cole conducted a study to assess the efficacy of a two-tier delivery system incorporating cationic RALA/pDNA nanoparticles (NPs) into a dissolvable MN patch for DNA vaccination against prostate cancer. The application of NP-loaded MN patches successfully resulted in the endogenous production of the encoded prostate stem cell antigen (PSCA). Furthermore, immunization with RALA/pPSCA-loaded MNs elicited a tumor-specific immune response against TRAMP C-1 tumors, postponed tumorigenesis in prophylactic models, with 1 mouse remaining tumor-free for 100 days after challenge. This provides additional evidence that this two-tier MN delivery system serves as a robust platform for prostate cancer DNA vaccination (Cole et al., 2019). In addition, Yan et al. devised a DNA vaccine encoding the secreted protein Ag85B of *Mycobacterium tuberculosis* and administered it in the skin using microneedles. This approach demonstrated an improvement in protective immunity compared to conventional intramuscular (IM) injection. Notably, MNs’ immunization was more effective in eliciting an antibody response than IM injection, particularly at a high dose of 12.6μg, which significantly decreased bacterial counts in both lungs and spleen compared to control groups. These findings suggest that the use of transdermal dissolving microneedles for DNA vaccination may offer a new strategy against tuberculosis (Yan et al., 2018). In another example, Huu Thuy Trang Duong et al. reported on the successful delivery of polyplex-based DNA vaccines using MNs arrays coated with a polyelectrolyte multilayer assembly of charge reversal pH-responsive copolymer and heparin. The charge reversal pH-responsive copolymer, consisting of oligo (sulfamethazine)-b-poly (ethylene glycol)-b-poly (amino urethane) (OSM-b-PEG-b-PAEU), served as a triggering layer in the assembly of the polyelectrolyte multilayer on microneedles. The unique charge reversal characteristics of this copolymer, specifically the OSM-b-PEG-b-PAEU copolymer, involve exhibiting a positive charge at low pH (pH 4.03) and transitioning to a negative charge when exposed to physiological pH conditions (pH 7.4). This property allows for the facile assembly and disassembly of polyelectrolyte multilayers. The electrostatic repulsion between heparin and the OSM-b-PEG-b-PAEU charge reversal copolymer initiated the release of DNA vaccines. *In vitro* studies demonstrated that DNA vaccines loaded onto microneedles were effectively transfected into RAW 264.7 macrophage cells, indicating the potential for these microneedles to deliver DNA vaccines to antigen-presenting cells. Moreover, vaccinating BALB/c mice with microneedles loaded with the DNA vaccine and coated with a polyelectrolyte multilayer induced robust, antigen-specific humoral immune response, the expression of Aβ was significantly elevated in mice treated with microneedles compared to those receiving subcutaneous injections, suggesting the potential effectiveness of this method for vaccination against diseases like Alzheimer’s (Duong et al., 2018b).

Dissolvable MNs technology encounters two primary challenges: the suboptimal transfection efficacy of pDNA after release from the microneedle matrix, and the constrained loading capacity of micron-scale devices. Two-tier delivery systems, which integrate microneedle platforms and DNA delivery vectors, have improved efficacy, yet the challenge of augmenting loading capacity persists. Grace Cole et al. employed lyophilization to augment the loading of RALA/pDNA nanoparticles within dissolvable PVA microneedles. *In vivo* delivery was substantially enhanced, reaching an appropriate range for DNA vaccination (∼50 μg per array) (Cole et al., 2018). To enhance the uptake of genetic material while ensuring biocompatibility and avoiding inflammatory responses or cytotoxicity associated with nondegradable synthetic polymers, Qu et al. utilized naturally-derived gelatin methacryloyl (GelMA) as a biodegradable polymer. They employed poly (β-amino ester) (PBAE) as a gene carrier for local and percutaneous controlled delivery of plasmid DNA (pDNA) *in vivo*, effectively penetrate the epidermal layer of the skin and reach the dermal layer, achieving a transfection rate of approximately 31% without causing skin damage (Qu et al., 2020).

**FIGURE 4:**
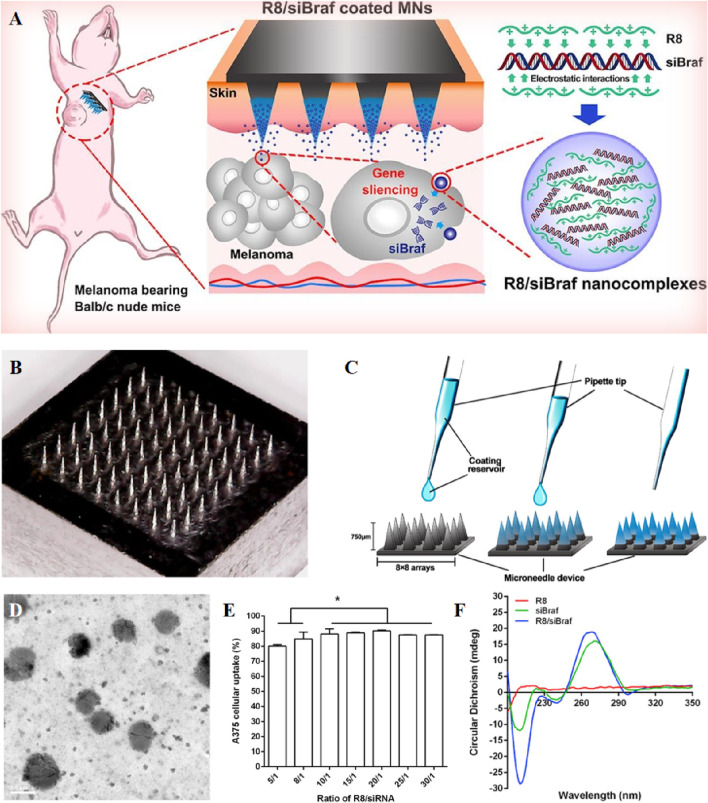
**(A)** Schematic representation of R8/siBRAF-coated MNs for targeted anti-melanoma therapy. **(B)** Image of stainless steel microneedles photographed by digital camera. **(C)** The coating process of R8/siBraf onto the microneedles. **(D)** Transmission electron microscope image of R8/siRNA nanocomplexes. **(E)** Cellular uptake of R8 and FAM-siRNA nanocomplexes at different N/P ratios by A375 cells after 4 h incubation. Each value represented mean ± standard derivation, *p < 0.05, n = 3. **(F)** Circular dichroism spectra of R8, siRNA, and R8/siRNA nanocomplexes. Reprinted with permission from Ref (Ruan et al., 2018).

Small interfering RNA (siRNA) in gene therapy offers relatively low toxicity and high specificity, attributed to its ability to target and silence specific genes (Figure 4) (Ruan et al., 2018). When delivered by MNs, siRNAs must be resistant to enzymatic degradation, capable of entering target cells, and able to escape the endosome–lysosome degradation axis. One solution to this challenge is to encapsulate these gene drugs in nanoparticles for transdermal delivery using MNs. Wang et al. introduced a nanoparticle-embedding MNs system featuring a soluble hyaluronic acid (HA) matrix. This innovative system utilizes a mesoporous silica (mSiO_2_) shell to both load and protect small interfering RNA (siRNA). Additionally, it incorporates an upconversion nanoparticle (UCNP) core for tracking microneedle skin penetration and the diffusion of nanoparticles. Studies showed an initial burst release of 75% of molecular beacons (MBs) within 12 h, followed by a sustained release, reaching 85% of MBs with 3 days (Wang et al., 2020). To introduce large molecule siRNA into tumor cells, Ruan et al. developed a siBraf delivery system based on cell-penetrating peptide octaarginine (R8) nanocomplexes combined with coated MNs. Specifically, they employed R8/siBraf-coated MNs (R8/siBraf coated MNs) for targeted anti-melanoma treatment, efficiency of A375 cellular internalization approached 90% (Ruan et al., 2018). The delicate and temperature-sensitive nature of mRNA drugs makes them unsuitable for delivery via MNs composed of metals, polymers, and ceramics. To overcome this challenge, Yu et al. employed frozen microneedles to deliver the mRNA vaccine, using subcutaneous injections of mRNA-loaded liposomes as a positive control. In the seventh week of the experiment, mouse serum was collected for an antibody neutralization assay, revealing that serum from the frozen microneedle group exhibited potent neutralizing activity against SARS-CoV-2 wild-type pseudovirus, with a virus inhibition rate surpassing 50% at a serum dilution of 1:320 (Yu et al., 2022).

**FIGURE 5:**
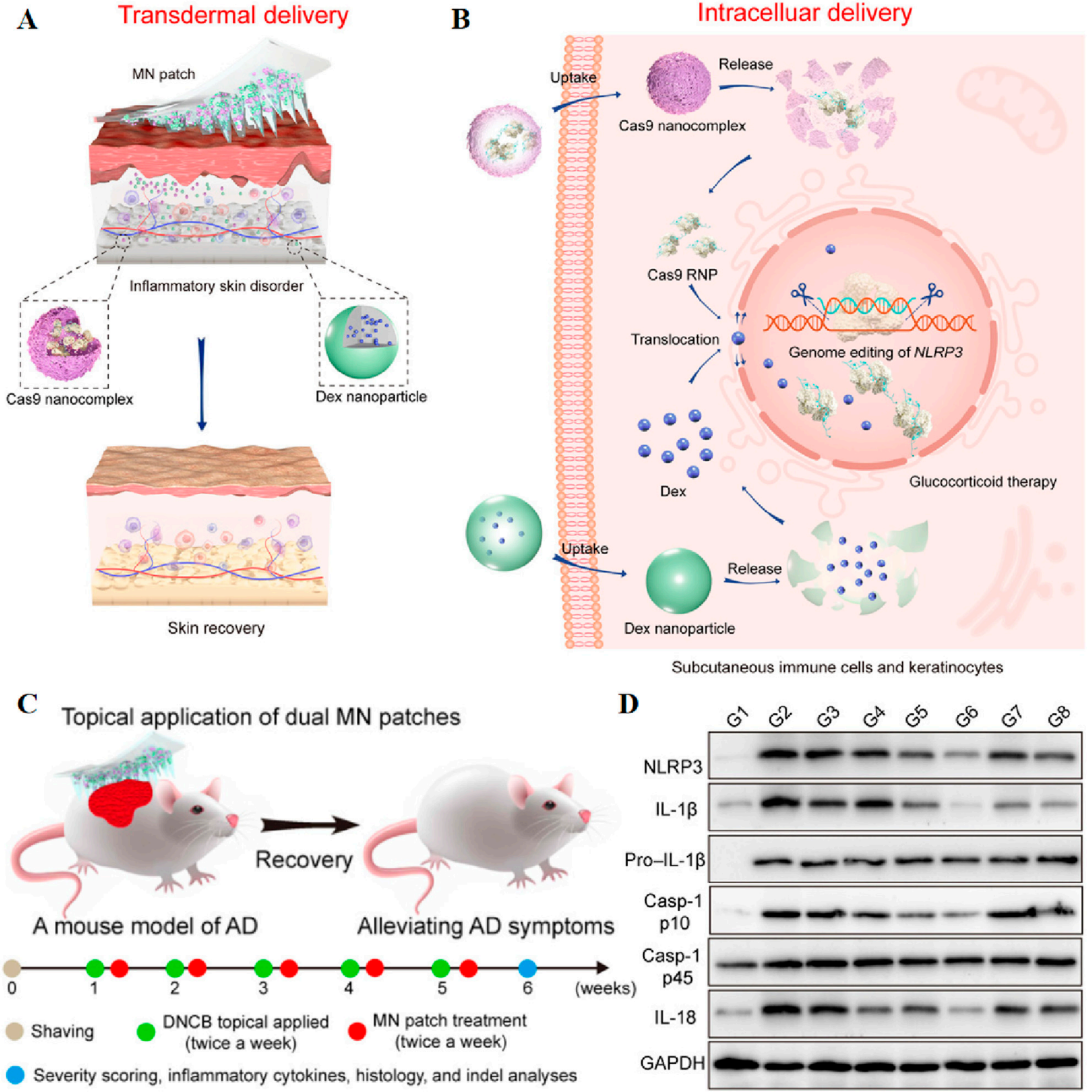
Schematic illustration of stepwise transdermal **(A)** and intracellular **(B)** delivery of genome-editing agents (Cas9) and glucocorticoids (Dex) for the treatment of ISDs. **(C)** Schematic illustration of dual MN patch for the treatment of DNCB-induced AD. **(B)** Photographs of mice treated with various formulations. **(D)** Immunoblot analysis of NLRP3 and other inflammasome protein expression in the dorsal skin homogenates. GAPDH, glyceraldehyde-3-phosphate dehydrogenase. Reprinted with permission from Ref (Wan et al., 2021).

In addition, MNs loading of ribonucleoproteins enables gene editing. For instance, Wan et al. reported a dissolvable MNs patch capable of mediating transdermal codelivery of CRISPR-Cas9-based genome-editing agents and glucocorticoids for the effective treatment of inflammatory skin disorders (ISDs) (Figure 5). Direct blockade of NLRP3 gene by CRISPR-Cas9 gene editing to reduce glucocorticoid resistance, the indel mutation frequency of the NLRP3 gene increased from 13.6% to 21.4% in the atopic dermatitis mouse model, and the expression levels of inflammatory cytokines IL-1β and IL-18 in skin tissues were significantly reduced. In the psoriasis model, the combination treatment increased the NLRP3 gene indel mutation frequency from 10.7% to 17.2% (Wan et al., 2021). The synergistic combination of gene therapy and photothermal therapy (PTT) has been extensively investigated as a promising strategy for cancer treatment. In this context, Xu et al. designed a MN patch co-loaded with p53 DNA and IR820, employing a two-step casting method for fabrication. Hyaluronic acid was selected as the matrix, and p53 DNA and IR820 were predominantly loaded into the tips to enhance utilization and minimize waste. The MN patch demonstrated efficient penetration of the stratum corneum and rapid dissolution, facilitating the release of p53 DNA and IR820 in the subcutaneous tumor site. The highly effective photothermal properties of IR820 led to a substantial temperature increase of 14.7 °C at the tumor site upon near-infrared light irradiation. The MN patch exhibited excellent antitumor effects, exhibited only 40% increase in tumor volume, highlighting the synergistic impact of gene therapy and PTT. The rapid release of genes is imperative upon the insertion of the MN patch (Xu et al., 2020). To achieve rapid gene release, Xinfang Li et al. capitalized on the acidic skin environment. They assembled pH-responsive polyelectrolyte multilayer (PEM) layers on the surface of polycaprolactone (PCL) microneedles, resulting in a rapid gene release of 33% in a simulated skin environment, compared to the 4% release observed with microneedles lacking PEM coatings (Li et al., 2019).

### 3.2 Chemical enhancers

**FIGURE 6:**
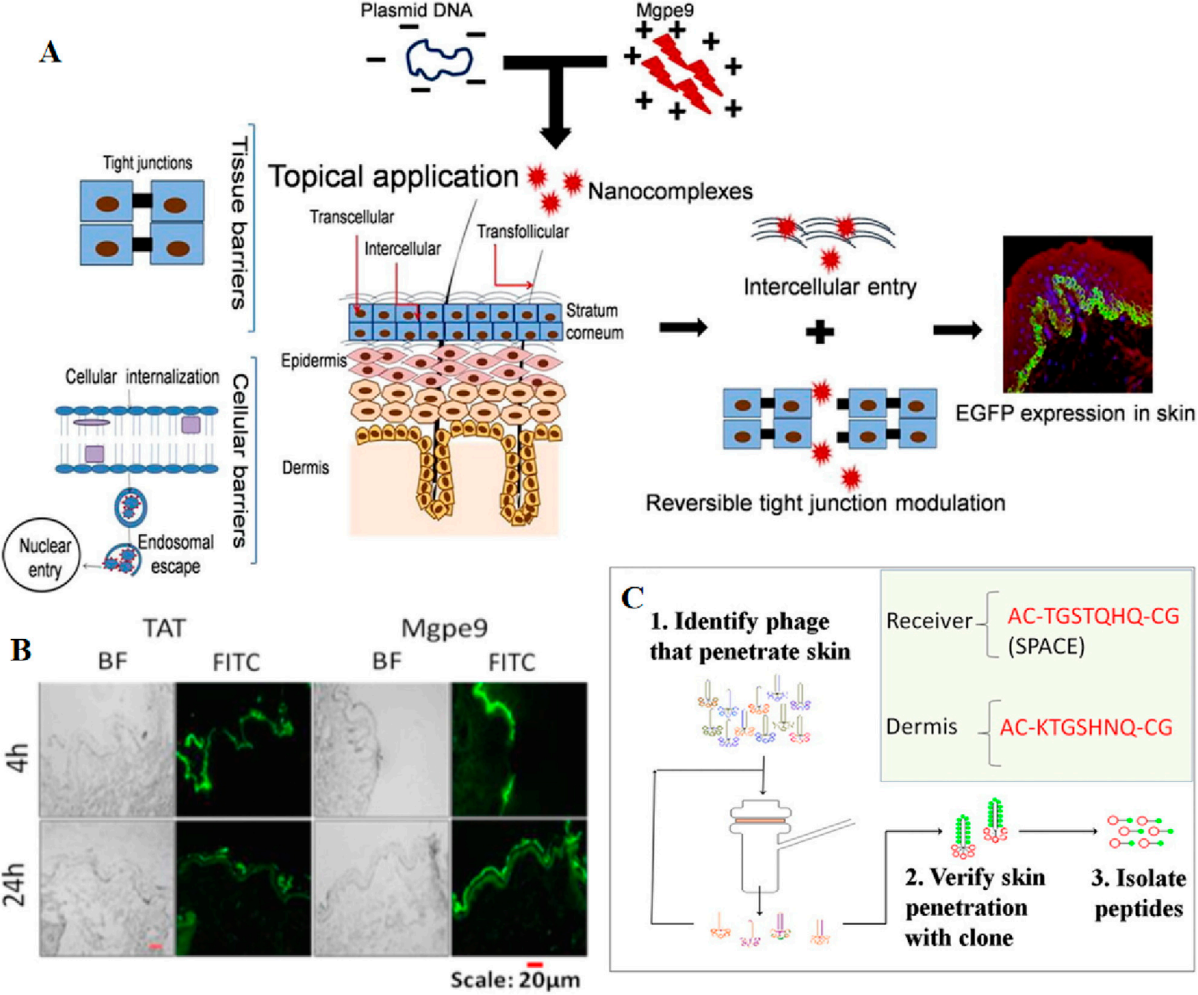
**(A)** Schematic representation of the process by which Mgpe9 peptide penetrates the skin for plasmid delivery. **(B)** Human skin penetration ability of FITC labeled peptides (Mgpe9 and TAT) 4 h and 24 h after application was studied using the peptide skin penetration test in independent experiments. Direct visualization of FITC fluorescence was performed after single topical application of peptides using fluorescence microscopy at ×10 magnification. Scale bar: 20 μm. Reprinted with permission from Ref (Wan et al., 2021). **(C)** A cell penetrating peptide, SPACE has been identified using *in vitro* phage display in porcine skin. Reprinted with permission from Ref (Hsu and Mitragotri, 2011).

Chemical enhancers have the capability of facilitating the penetration of drugs through the stratum corneum into the skin through a chemical process (Marwah et al., 2016). Peptides, whether natural or synthetic, possess superior biocompatibility. Among them, cell penetrating peptides possess unique ability to directly penetrate the cell membrane without interfering with its structure, have recently gained attention as potential vectors for macromolecules, including proteins and plasmid DNA. A team led by Manika Vij et al. identified an amphotericin M peptide (CRRLRHLRHHYRRRWHRFRC), referred to as Mgpe9, which possesses the unique ability to permeate both *in vitro* and *in vivo* skin without any external intervention. This peptide demonstrates the capability to deliver plasmid DNA into skin cells and holds potential as a nucleic acid transporter (Figures 6A, B) (Vij et al., 2016). As biological macromolecules, naked siRNAs exhibit low target cell permeability when delivered transdermally. To address this issue, Tamae UCHIDA et al. employed two peptides, AT1002 and Tat, as chemical osmotic enhancers to facilitate siRNA delivery. The results show that these peptides significantly accelerated the transdermal delivery of siRNA and approximately 60% of the siRNAs retained stability in 10 h after the addition of RNaseA (Uchida et al., 2011). Furthermore, two articles discovered that the SPACE-peptide can enhance siRNA penetration across the stratum corneum into the epidermis and dermis, and can augment the penetration of various cell types (Figure 6C) (Chen et al., 2014; Hsu and Mitragotri, 2011). Building on this foundation, Ming Chen et al. combined the SPACE peptide with the DOTAP-based proteasome vector system to enhance siRNA delivery. Ultimately, the results confirmed that the SPACE peptide enhances the skin penetration of siRNA, and this skin penetration was further enhanced when combined with the DOTAP proteasome (Chen et al., 2014). Using a unique mouse model, Vikas Hegde et al. compared the effectiveness of various nucleic acid gene-silencing agents in the skin of living animals. They discovered that only the commercially available “self-delivery” modified accell-sirna (Dharmacon) demonstrated potent and durable gene silencing *in vivo*. Consequently, they developed a novel topical formulation, and sustained N40% luc2p inhibition was observed after just two 1-hour treatments with accell-sirna in the formula, successfully delivering siRNA locally (Hegde et al., 2014).

Chemical enhancers can also be combined with iontophoresis to facilitate transcutaneous gene delivery. Liu et al. used a model anion drug, limonene/ethanol, which resulted in a slight increase in the penetration of sodium fluorescein. It was demonstrated that chemical osmosis agents combined with iontophoresis could increase the delivery of endothelial oligonucleotides, but it still could not achieve complete delivery (Liu et al., 2013). The use of chemical enhancers faces several challenges, such as interfering with drug activity (Lv et al., 2006), limited potency, irritation caused by potent enhancers, unpredictability of action, and complex mechanisms. These issues need to be addressed in the future development of chemo-osmotic agents (Kováčik et al., 2020).

### 3.3 Ultrasound

The use of ultrasound in transdermal drug delivery is a time-honored approach that dates back to the middle of the 20th century (Mitragotri, 2013). Sonophoresis refers to the transdermal drug delivery using ultrasound waves to penetrate through the skin membrane. Ultrasound-induced microfluidics and cavitation-induced pressure perturb the lipid structure of the stratum corneum, and the thermal effect of ultrasound induces localized skin warming to improve blood circulation, leading to the dilation of pores and sweat glands, and expedite the absorption of therapeutic agents (Phatale et al., 2022). In transdermal drug delivery, both low-frequency sonophoresis (LFS) and high-frequency sonophoresis (HFS) are commonly employed (Polat et al., 2011). The expansion of the void in SC induced by LFS has been experimentally demonstrated, leading to an enhanced drug penetration (Paliwal et al., 2006). Sonophoresis typically employs ultrasound ranging from 4 kHz to 5 MHz, directly applied to the skin. This technique facilitates drug delivery by enhancing the permeation of the skin barrier (Zhao et al., 2023).

Compared with other transdermal techniques, ultrasound offers advantages such as deep tissue and organ penetration, high biological safety, and portable equipment (Zhao et al., 2023). Utilizing ultrasound, Schoellhammer et al. successfully administered mRNA and siRNA to the colonic mucosa of mice. This resulted in the downregulation of target mRNA expression, showcasing the potential application of ultrasound for delivering nucleic acids to the gastrointestinal tract (Schoellhammer et al., 2017). Dominic M. Tran et al. employed ultrasound-mediated gene delivery (UMGD) of nonviral vectors to target the liver in large animal models. Successful UMGD relies on acoustic perforation induced by exogenous cavitated nuclei, such as microbubbles, which oscillate radially under specific frequencies and peak negative pressures (PNPs). This phenomenon leads to transient pores formation in the cell membrane and opening of endothelial tight junctions. Nonviral vectors, including bare plasmid DNA (pDNA) carrying the gene of interest, are effectively disseminated during this transient state (Tran et al., 2019). Despite the proven efficacy of ultrasound in enhancing transdermal drug delivery, achieving precise control over the location, shape, and size of the localized skin delivery area remains challenging. To address this issue, Hu et al. developed an ultrasonic erosion protocol to establish precise delivery zones in mouse skin, facilitating the successful delivery of nanoparticles (India ink) and hepatitis B antigen. The importance of hair follicles in ultrasound-mediated transdermal delivery is also illustrated (Hu et al., 2019).

**FIGURE 7:**
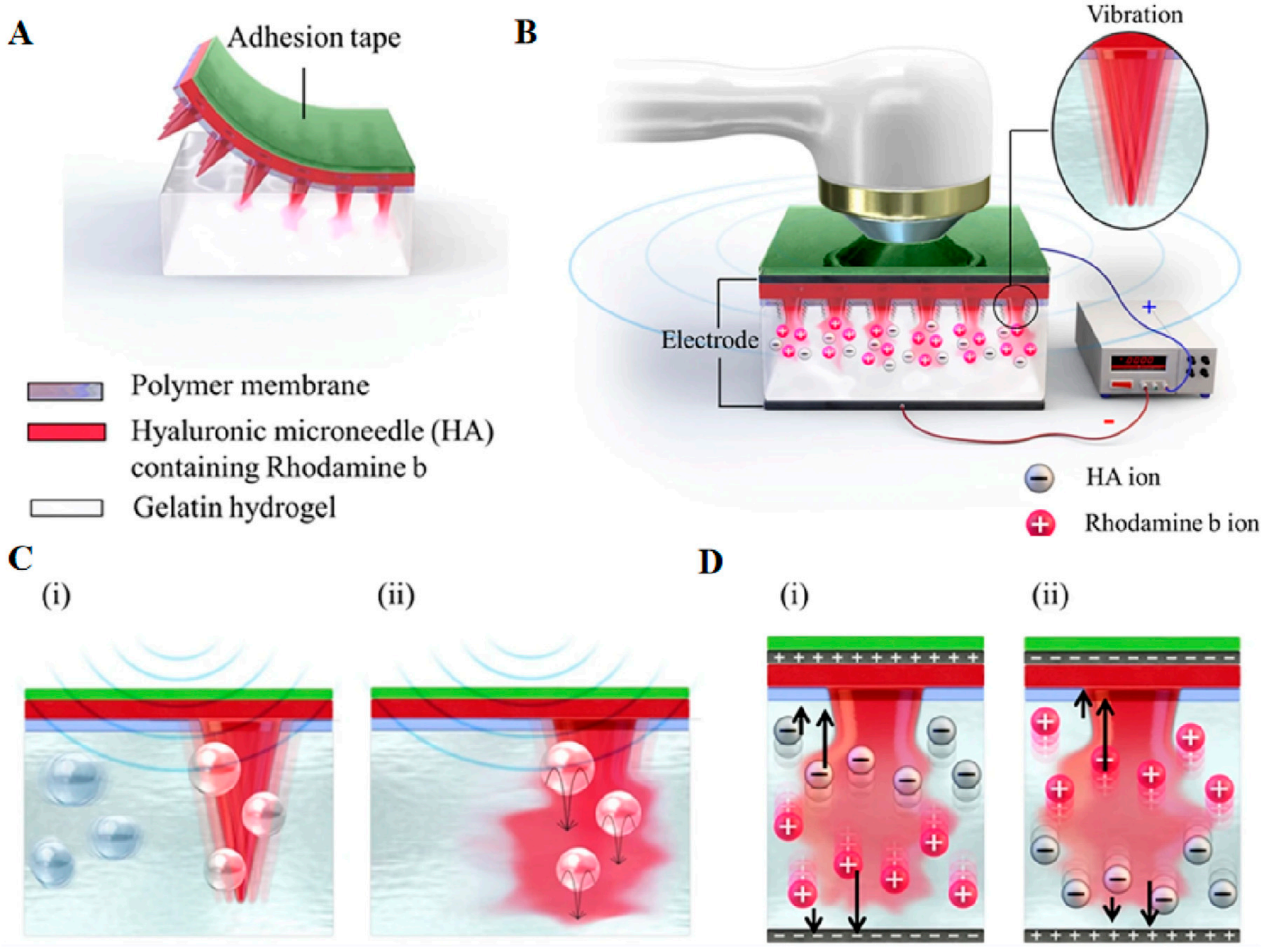
**(A)** The attachment structure of HA microneedles in gelatin hydrogel, **(B)** multi-system structure of HA microneedles, and the ultrasonic and electric field in gelatin hydrogel. The inset image indicates the vibration of the needle. Schematic representation of the predicted scenario of **(C)** the dissolution mechanism using ultrasound; (i) fine bubbles and the vibration of the needles by ultrasound and (ii) needle dissolution by ultrasound, **(D)** The predicted dissolution mechanism and ion direction in relation to the direction of the electric field; (i) voltage > 0 and (ii) voltage < 0. Reprinted with permission from Ref (Bok et al., 2020b).

In recent years, bubble-assisted ultrasound has been gradually applied (Escoffre et al., 2016). However, the impact of microbubbles generated during ultrasound stimulation on drug transduction to tumor cells remains not obvious. Zandi et al. have developed an electrochemical stimulator integrated on zinc-oxide nanowires modified microneedles for the localized intratumoral generation of microbubbles (MBs). A ZnO nanowire-based microbubble generator probe was employed to induce cavitation, thereby enhancing ultrasound-assisted drug delivery efficiency to the tumor. This approach can reduce the side effects of chemotherapy and improve the efficacy of chemotherapy (Zandi et al., 2019). In addition, ultrasound can be combined with other transdermal drug delivery methods (Schoellhammer et al., 2014), such as MNs (Bok et al., 2020a; Chen et al., 2010; Ryu et al., 2018) and iontophoresis (Park et al., 2019). MNs penetrate the cuticle to deliver drugs to the epidermis, while acoustic phoresis emitters can enhance macromolecular diffusion rates by providing energy and inducing acoustic cavitation. Furthermore, the application of ultrasound has been shown to facilitate the dissolution of hyaluronic acid (HA) microneedles (Figure 7) and enhance drug permeation (Bok et al., 2020a). The combination of MNs and low-frequency ultrasound has been demonstrated to improve the transdermal drug delivery rate *in vitro* and enable the transdermal delivery of macromolecular drugs such as calcein and bovine serum albumin (BSA) (Chen et al., 2010). Yeong Chae Ryu et al. penetrated pig skin using Cy3-labeled siRNA, MNs, and ultrasound, resulting in a 6.9-fold higher concentration of siRNA penetration than microneedles alone. Similarly, they observed a 5.2-fold higher penetration of ovalbumin than microneedles alone when employing Cy3-labeled ovalbumin, microneedles, and ultrasound (Ryu et al., 2018). Park et al. have developed a novel device capable of simultaneous acoustic and ion electrophoresis, enabling enhanced skin penetration of multiple drugs with reduced intensity and application time, thereby mitigating skin irritation (Park et al., 2019).

Despite numerous studies investigating the efficacy and safety of ultrasound-mediated transdermal drug delivery, certain limitations remain to be addressed pertaining to modifiable parameters including frequency, duty cycle, coupling medium, and pressure amplitude. The potential of ultrasound-mediated transdermal drug delivery is promising. With an enhanced comprehension of this technology by physicians, engineers, and scientists coupled with their persistent efforts, ultrasound-based transdermal drug delivery platforms are poised to advance closer towards clinical application (Seah and Teo, 2018).

### 3.4 Iontophoresis (IP)

IP is a skin-permeation technology that employs low-level electrical current to solubilize drugs and facilitate their transdermal delivery to the underlying tissues of the skin (Meadows et al., 2014). IP uses electric fields to deliver drugs and vaccines to the skin, is particularly effective against charged and polar molecules (Banga et al., 1999). Two electrode patches form circuits through the skin that disrupt the lipid arrangement between stratum corneum cells and reversibly modifying the skin’s barrier properties by applying a small electric current to the skin. This process enhances skin permeability, facilitating the passage of therapeutic agents through the skin barrier (Hettinga and Carlisle, 2020). IP applications are simple and do not require complex devices (Paudel et al., 2010). In addition, IP does not produce cytotoxicity and is easy to combine with other drug delivery methods (Hasan et al., 2022). Studies indicate that no obvious skin changes are observed at current densities below 10 mA/mm^2^. In each application, the amplitude of the current usually below 0.5 mA/cm2, which is considered physiologically acceptable (Hasan et al., 2022). Nevertheless, it is essential to account for individual differences in practice. Careful selection of treatment parameters, along with close monitoring of the skin response during treatment, is necessary.

**FIGURE 8:**
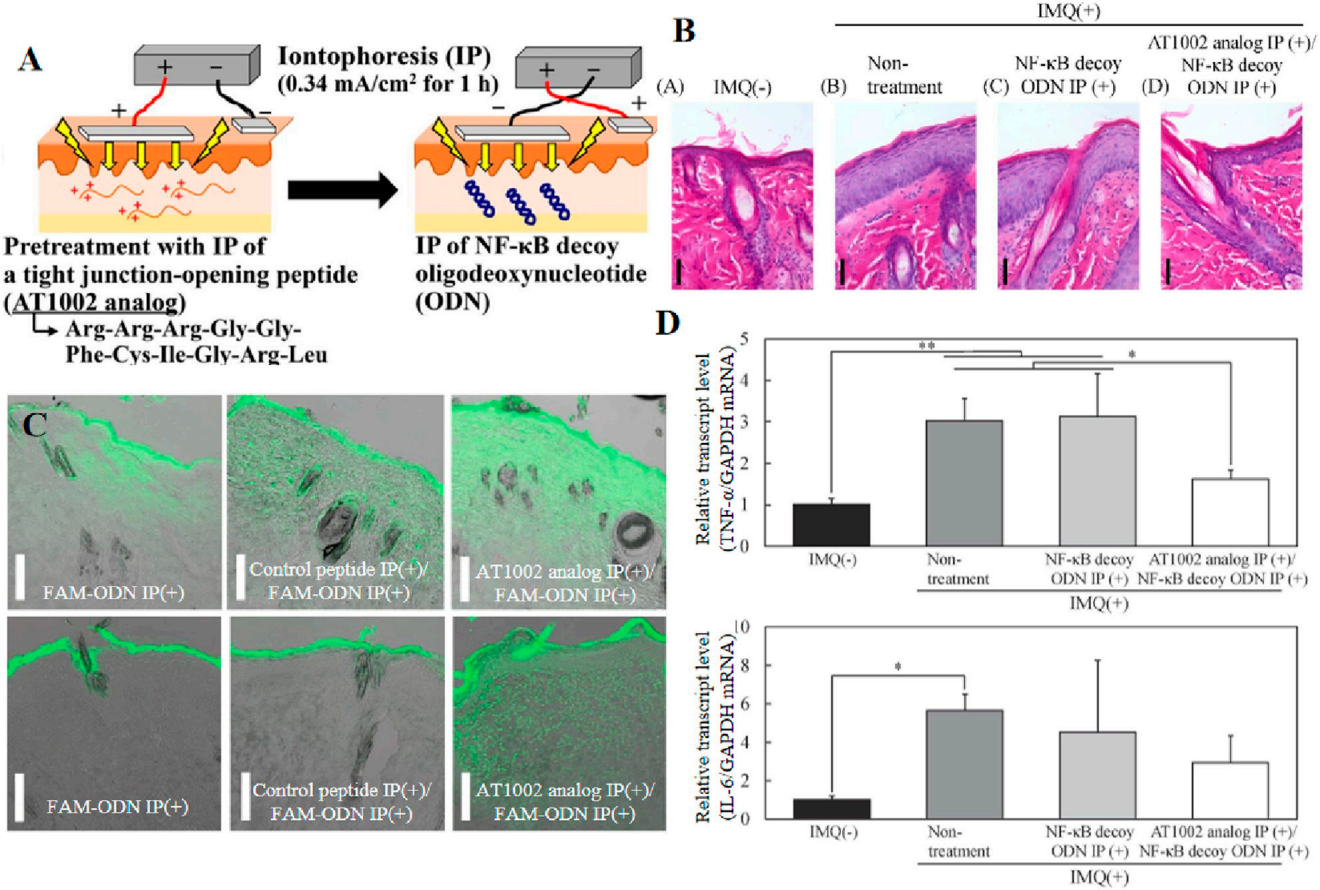
**(A)** Schematic of the iontophoretic technique. **(B, C)** Intradermal distribution of IP-administered fluorescence-labeled oligodeoxynucleotides in healthy and psoriatic skin. **(D)** TNF-α and IL-6 mRNA levels in the skin of rats after IMQ treatment of a psoriasis model. Reprinted with permission from Ref (Fukuta et al., 2021).

In recent years, IP has been used to deliver siRNA, CpG and mRNA. The delivery of biomacromolecules through IP can effectively treat inflammatory skin diseases. For example, the study conducted by Fukuta et al. demonstrated the successful delivery of IP-delivered antibodies to inflamed tissues, leading to a significant improvement in epidermal hyperplasia in rats with silver cuttle disease. This groundbreaking finding establishes IP as a non-invasive and efficient intradermic drug delivery method (Fukuta et al., 2020). One year later, Tatsuya Fukuta al. Combined AT1002 and IP to deliver NF-κB decoy oligonucleotides, and promoted macromolecular transfer through the synergic effect of weak current mediated intercellular junction cutting and AT1002s tight junction opening ability, thus achieving stronger intercellular space cutting and overcoming the skin barrier of psoriasis thickening. In addition, NF-κB decoy oligodeoxynucleotides play a therapeutic role in the intradermal transmission, and significantly inhibit the upregulation of the mRNA level of inflammatory cytokines induced by psoriasis, thus improving epidermal hyperplasia (Figure 8) (Fukuta et al., 2021). Additionally, K. Kigasawa et al. employed iontophoresis technology to selectively accumulate siRNA in the epidermis while avoiding dermal deposition. This approach achieved effective delivery without inducing tissue damage and successfully suppressed the expression of endogenous immunomodulatory cytokines (Kigasawa et al., 2010).

IP technology has extended to the field of cancer treatment. Jose et al. encapsulated curcumin in cationic liposomes and then complexed it with STAT3 siRNA to study the transdermal effect of co-delivery of curcumin and STAT3 siRNA by liposomes in pig skin models *in vitro* through non-invasive local ion implantation. Local iontophoresis enhances the skin penetration of the nanocomplex, allowing it to penetrate the living epidermis for non-invasive ion delivery of curcumin and siRNA for skin cancer treatment (Jose et al., 2017). The literature also includes numerous reports on IP-administered drugs utilized in the treatment of melanoma. Kaoru Kigasawa et al. investigated the antitumor activity of CpG-ODN (unmethylated cytosine phospho-guanosine oligodeoxynucleotides) delivered by iontophoresis for mouse B16F1 melanoma immunotherapy. Ultimately, iontophoresis-delivered CpG-ODN induced both local and systemic immune responses, significantly inhibiting melanoma growth after repeated ion permeation (Kigasawa et al., 2011). Husseini et al. prepared a minimal mRNA vaccine encoding the tumor-associated antigen human gp10025-33 peptide (KVPRNQDWL) for potential melanoma therapy. The delivery of the iontophoresis-induced mRNA vaccine induces an immune response that activates skin-resident immune cells (Husseini et al., 2023). In addition, elevated mRNA expression levels of various cytokines, primarily interferon (IFN)-γ, and infiltration of cytotoxic CD8^+^ T cells in tumor tissues have been observed in mice with melanoma. In addition, Suman Labala et al. delivered layer-by-layer gold nanoparticles (LbL-AuNP) via IP as a carrier for ion delivery of STAT3 siRNA for melanoma treatment (Labala et al., 2016).

Many factors affect the effectiveness of IP, including the properties of the drug itself such as the pH of the drug solution, the size of the drug molecules, and the hydrophilicity of the drug. Electrode type, current intensity, application time, current type, etc (Akhtar et al., 2020). The successful delivery of 15-kDa macromolecules is limited by IP. Achieving effective IP-mediated transdermal penetration and therapeutic concentrations of such macromolecules poses challenges. Furthermore, the transfer efficiency may vary based on the physicochemical properties (e.g., solubility, stability) of each macromolecule. The application of IP can also have potential effects on the skin, leading to skin irritation, numbness, itching, and erythema in patients. Additionally, improper electrode selection or placement on damaged skin, prolonged usage duration, and excessive current density may increase the risk of burns (Hasan et al., 2022). Most commercially available IP devices are expensive, bulky, and require an external power supply (Yamada and Prow, 2020). Therefore, there is a need for intelligent IP devices with high cost-performance. The clinical application of IP for these drugs is still at the laboratory level, moreover. There are several challenges that still need to be overcome for the clinical translation and commercialization of biomacromolecule IP. A good option to improve it may be making IP work in conjunction with other enhancement techniques (Hasan et al., 2022).

### 3.5 Electroporation

Electroporation, an innovative transdermal delivery method, transiently rearranges lipid molecules to create reversible hydrophilic pores in the cell membrane by subjecting it to high pressure and short-time electrophoresis, enabling the transport of large molecules (Ita, 2016). Normally, transient water pores that are thermodynamically unstable in cell membranes also appear in the absence of external electric field stimulation, which can only be maintained for nanoseconds. However, when the cell membrane is exposed to an electroporation electric field, it can change the permeability of cell membranes (Gong et al., 2022). Electroporation of the skin is a temporary phenomenon that occurs when an applied electric field surpasses its critical transmembrane potential. Electroporation, as a prevalent cell transfection strategy, possesses numerous merits. Firstly, it is a straightforward and efficient method. Secondly, it obviates the need for vectors, thereby circumventing interference with the immune system. Thirdly, in contrast to other permeation techniques, electroporation does not breach the cell membrane; instead, it transiently enhances permeability. In addition, electroporation offers the advantage of controlling the membrane disruption effect by adjusting parameters (Gong et al., 2022). However, precise mechanisms governing alterations in skin structure during perforation remain undefined (Ita, 2016).

Traditional skin electroporation poses a risk of potential skin damage owing to its high voltage. In addressing this concern, Huang et al. implemented a strategy involving the integration of a microneedle roller and a flexible cross-finger electroporation array (FIEA) to facilitate the efficient delivery of DNA and siRNA into the skin of mice. This innovative approach allows for the successful transportation of nucleic acids at lower voltages, concurrently exhibiting favorable safety profiles (Huang et al., 2050). Cindy Bernelin-Cottet al comparatively evaluated the efficiency of vaccine DNA delivery *in vivo* to pigs using dissolvable microneedle patches, intradermal inoculation with (ID), surface electroporation (EP), with DNA associated or not to cationic poly-lactic-co-glycolic acid nanoparticles (NPs). They used a luciferase encoding plasmid (pLuc) as a reporter and vaccine plasmids encoding antigens from the Porcine Reproductive and Respiratory Syndrome Virus (PRRSV), a clinically-significant swine arterivirus. This study concludes that successful DNA vaccine administration in skin can be achieved in pigs with electroporation and patches, but only the former induces local inflammation, humoral and cellular immunity, with the highest potency when NPs were used. This finding shows the importance of evaluating the delivery and immunogenicity of DNA vaccines beyond the mouse model in a preclinical model relevant to human such as pig and reveals that EP with DNA combined to NP induces strong immunogenicity (Bernelin-Cottet et al., 2019).

## 4 Transpulmonary gene delivery system

Due to the extensive surface area, abundant vascular network, ease of administration through inhalation, the respiratory tract represents an optimal non-invasive route for the gene delivery (Gomes Dos Reis et al., 2017), offering distinct advantages in terms of targeted therapeutic effects on diseased organs and minimal toxicity to target cells (Bardoliwala et al., 2019). In the realm of lung gene therapy, the effective delivery of nucleic acid cargo to specific target areas within the lungs faces hindrances from various factors, including lung architecture, clearance mechanisms, immune activation, the presence of airway mucus, and the absence of representative biological models (Birchall, 2007). Delivering therapeutic genes to the pulmonary system through inhalation requires the administration of biologic drugs in aerosol form, a process that involves intricate formulation engineering and manufacturing processes (Chow et al., 2021). Developing effective aerosol formulations is crucial for advancing lung gene therapy, particle size dictates the deposition site of the agents within the lungs, and a biocompatible aerosol with high stability can ensure the efficiency in pulmonary gene therapy, reducing irritation and toxicity to the respiratory tract and lungs. When choosing the most suitable approach for delivering nucleic acids into the lungs, gene delivery presents three fundamental options: aerosolization of liquid-suspended gene particles, aerosolization of dry formulations of gene vectors with carrier particles, or pressurized ejection of DNA from propellant dispersions (Table 3). Among these methods, atomization stands out as the predominant technique for introducing gene vectors into both animal and clinical studies (Birchall, 2007).

**TABLE 3:** Summary of different delivery methods for gene delivery to the lung.

Strategies of administration	Gene	Delivery vectors	Applications	Evaluation models	Refs
Nebulizer	IVT-mRNA	Lipoplexes	Lung-related and respiratory diseases	16HBE14o- (16HBE)	Guan et al. (2021)
Aerosol inhalation	IVT-mRNA	Hyperbranched poly (beta amino esters)	Lung epithelium delivery system	C57BL/6 female mice	Patel et al. (2019)
Nebulizer	mRNA	Poly-beta amino ester	Respiratory virus infections	Golden Syrian hamster	Vanover et al. (2022)
Nebulizer	siRNA	Nanogel coated with Curosurf^®^	Pulmonary disease	H1299_eGFP cells	Merckx et al. (2020)
Intratracheal	DNA	Poly (β-amino esters)	Lung gene therapy	Balb/C mice	Mastorakos et al. (2015)
Supercritical fluid technology	siRNA	Poly-L-lactide porous microparticles	Cancers with multidrug resistance	Human small cell lung cancer cells	Xu et al. (2018)
Spray drying	siRNA	Lipid nanoparticle (PEG-DMG)	Respiratory diseases	H1299-GFP cells	Zimmermann et al. (2022)
Spray drying	bDNA	Polyethylenimine	Pulmonary diseases	A549 cells	Keil et al. (2019)
Inhalation powder	pDNA	Naked pDNA	Pulmonary gene therapy	ICR female mice	Ito et al. (2019a)
Inhalation powder	siRNA	Naked siRNA	Respiratory epithelium disease	ICR female mice	Ito et al. (2019b)
Spray drying	mRNA	Lipid nanoparticle	Gene delivery	Male Wistar Han rats	Friis et al. (2023)
Spray drying	siRNA	DOTAP modified PLGA	Respiratory disease	EGFP-H1299 cells	Jensen et al. (2012)
Pressurized metered dose inhalers	siRNA	G4NH2-TPP	“non-druggable” diseases	EGFP-A59 cells	Bielski et al. (2017)
Pressurized metered-dose inhalers	DNA	Cationic-polymer	Lung gene therapy	A549 cells	Conti et al. (2012)

### 4.1 Nebulized formulations

**FIGURE 9:**
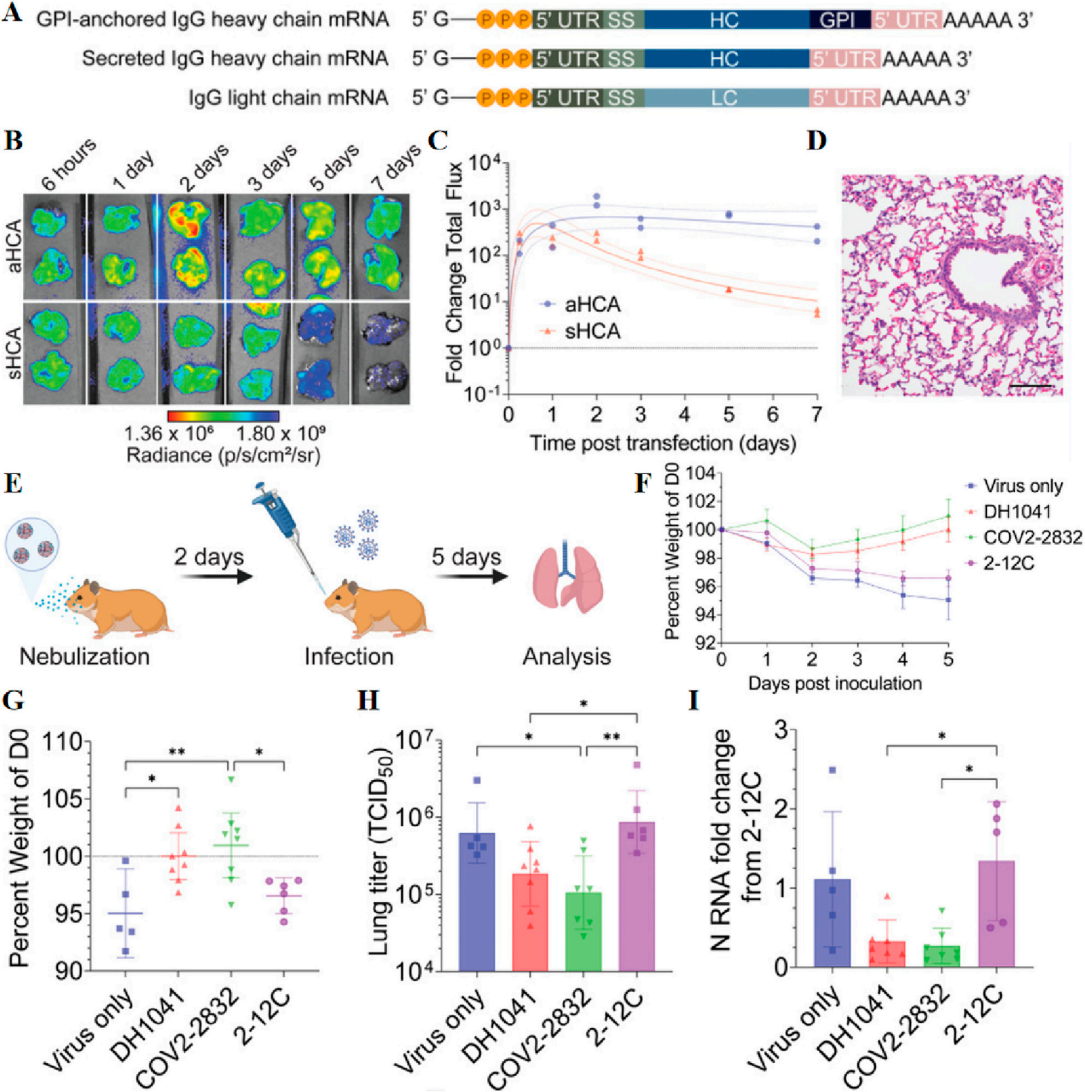
**(A)** mRNA schematics encoding GPI-anchored and secreted IgG heavy chains and the IgG light chain sequences. **(B)** Luminescence imaging of the lungs of nebulized mRNA-treated hamsters. **(C)** Quantification of luminescence in lungs. **(D)** Hamster lungs were excised and assessed for pathology via H&E staining at 24 h post-transfection of 31 µg of aNLuc mRNA. **(E)** Schematic representation of the preventive efficacy of GPI-anchored anti-SARS-CoV-2 antibodies in hamsters tested for aerosolized mRNA expression. **(F)** Mean hamster weights over time normalized to the day of infection. Bars indicate mean ± SEM. **(G–I)** Individual hamster weights **(G)**, lung titer **(H)**, and lung normalized viral N RNA **(I)** on day 5 post infection. Reprinted with permission from Ref (Vanover et al., 2022).

Nebulisation refers to the process of dispersing a liquid into fine droplets, which can be inhaled (Chow et al., 2021). Nebulizers are employed for generating liquid aerosols and can be used to administer significant quantities of drug solutions or suspensions through inhalation. They are often preferred in cases where measurement is unsuitable for a mixture inhaler or for measuring inhaler drugs (Lam et al., 2012). Additionally, nebulizers could be considered for the delivery of siRNA and mRNA. Daryll Vanover et al. have developed an inhalable formulation that facilitates the expression of membrane-anchored neutralizing antibodies encoded by mRNA in the lungs, effectively mitigating SARS-CoV-2 infection. This study demonstrates that nebulizer-based delivery of these mRNA-expressed neutralizing antibodies proves highly effective in eliminating the disease (Figure 9) (Vanover et al., 2022). Musa Khaitov et al. have developed a suction-based modified siRNA-peptide tree macromolecular preparation for COVID-19 treatment strategies (Khaitov et al., 2021). However, the delivery of siRNA drugs to cells is inefficient (Merckx et al., 2020; Merckx et al., 2018) and requires overcoming numerous extracellular and intracellular barriers by siRNA-loaded nanoparticles (De Backer et al., 2015). To address this concern, hybrid nanoparticles have been developed, consisting of nanogels with loaded siRNA cores and coated with clinically approved pulmonary surfactants. Importantly, the endogenous protein pulmonary surfactant-B has shown potential as an intracellular siRNA delivery enhancer (Merckx et al., 2018).

To enable the long-term preservation of siRNAs, the team successfully lyophilized lung surfactant-coated siRNA-loaded nanogels (siNGs) without the need for freezing or cryoprotectants. Following freeze-drying, reconstitution, and atomization processes, the physicochemical properties of the nanocomposites remained unchanged, while retaining the ability of nanocellulose to efficiently deliver siRNA into the cytoplasm of human lung epithelial cell lines (Merckx et al., 2020). Additionally, Lynn De Backer et al. have proposed the potential co-administration of siRNA-loaded nanoparticles (NPs) alongside pulmonary surfactants (De Backer et al., 2015). Whereas transpulmonary gene delivery fails to achieve sustained high levels of transgene expression *in vivo*, Mastorakos et al. have successfully attained stable and elevated levels of transgene expression for a minimum duration of 4 months following a single administration of poly (β-amino esters) (PBAEs) slime penetrating DNA nanoparticles (Mastorakos et al., 2015).

The utilization of *in vitro* transcribed (IVT) mRNA exhibits extensive therapeutic applicability due to its ability to regulate the temporal and dose-dependent expression of encoded proteins (Guan et al., 2021; Patel et al., 2019). Patel et al. synthesized hyperbranched poly (beta amino esters) (hPBAEs), achieving a polymer nano formulation with a stable concentration suitable for efficient pulmonary delivery. Repeated inhalation of hPBAE-mRNA resulted in consistent production of lung proteins, without any observed local or systemic toxicity (Patel et al., 2019). Guan et al. have successfully formulated liposomes containing *in vitro* transcribed mRNA encoding alpha-1-antitrypsin (A1AT-mRNA), which can be efficiently transfected into human bronchial epithelial cells without any observed toxicity. The resulting A1AT produced by atomized A1AT-mRNA lipid plexus-transfected cells demonstrates functional properties, effectively inhibiting the activities of trypsin and elastase (Guan et al., 2021). To overcome the low membrane permeability of pDNA, Gomes dos Reis et al. employed a cell-penetrating peptide as an uptake enhancer for pDNA delivery to the lungs, forming a complex between pDNA and CPP. The proposed approach effectively addresses the challenges associated with pDNA degradation caused by inadequate protection (Gomes Dos Reis et al., 2018).

The presence of cystic fibrosis (CF) sputum poses a challenge to the local delivery of gene transfer agents to the lungs. To overcome this physical barrier, Ibrahim et al. employed mannitol microparticles as a vehicle for gene transfer agents, creating a local osmotic gradient (Ibrahim et al., 2011). Furthermore, the negative impact of bacterial lung infection on atomization efficiency in cystic fibrosis gene therapy was addressed by introducing a novel type of lipid chain n-heterocyclic carbon silver complexes with cationic lipid and DNA (ternary combination). These complexes were successfully delivered via atomization for therapeutic genes targeting lung infections (Mottais et al., 2019). Nebulisation is also utilized for vaccination purposes. The bacterial culture supernatant extract (CSE) anthrax vaccine, developed by Li-Na Zhai et al., in liquid, powder, and powder formulations, can be administered via aerosol intratracheal inoculation, making it suitable for pulmonary anthrax immunization through inhalation (Zhai et al., 2023).

### 4.2 Dry powder formulations

**FIGURE 10:**
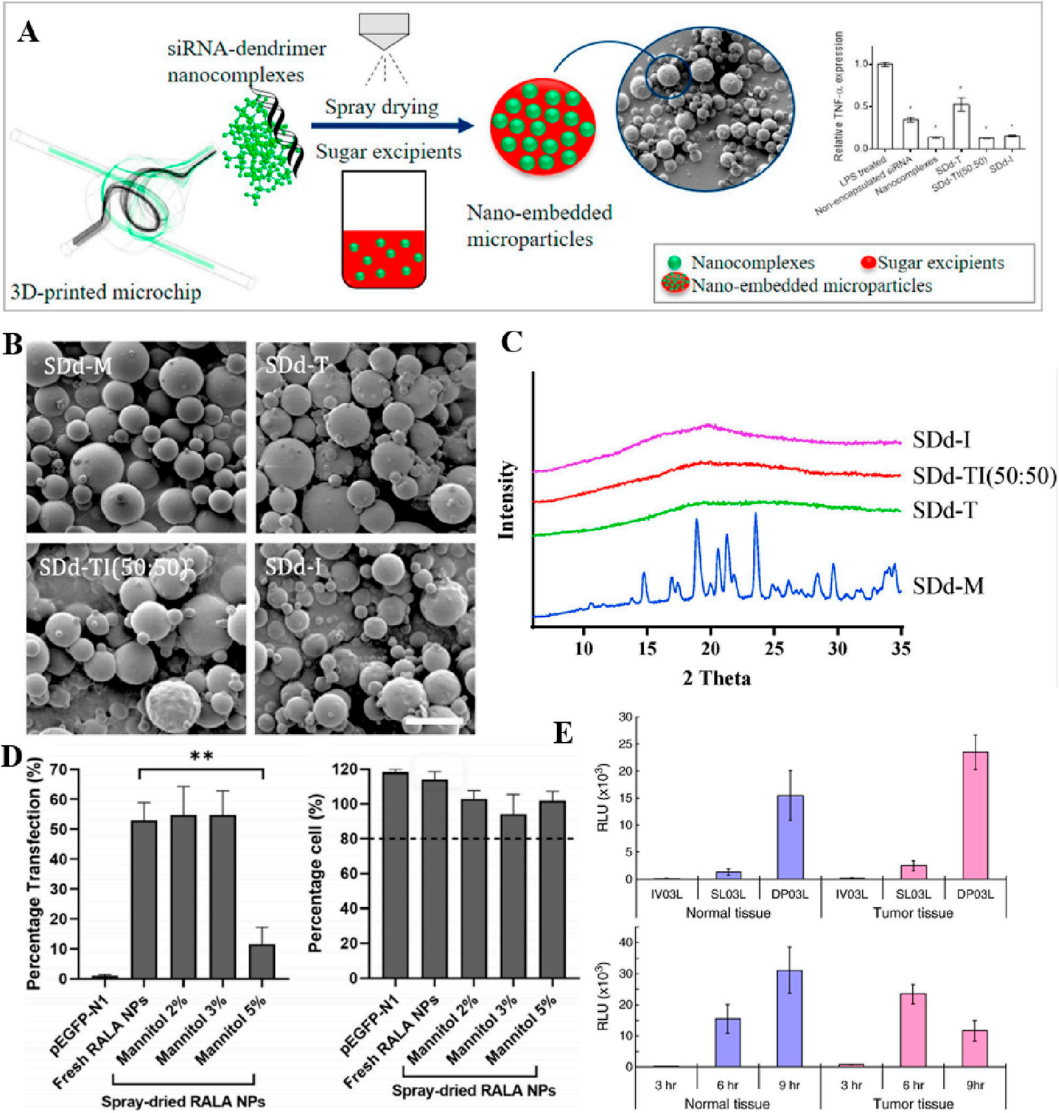
**(A)** Schematic representation of siRNA-dendritic nanocomposites prepared by 3D printed micromixers processed into dry powder. **(B)** Representative SEM images of spray dried NEMs. **(C)** XRPD patterns of NEMs prepared using different excipients. Reprinted with permission from Ref (Agnoletti et al., 2017). **(D)** *In vitro* transfection efficiency of spray-dried RALA/pEGFP-N1 complexes at different mannitol concentrations. Reprinted with permission from Ref (Munir et al., 2022). **(E)** Luciferase expression in normal (N) and tumorous (T) tissues in the mouse lung burdened with pulmonary metastasis. Reprinted with permission from Ref (Okamoto et al., 2011).

Due to its simplistic formulation, nebulization remains the preferred treatment option (Chow et al., 2021). However, inhaled powder formulations exhibit significantly reduced susceptibility to instability, chemical contamination, and microbial contamination compared to liquid formulations (Carneiro et al., 2023). Dry powder inhalation route is widely regarded as the optimal choice for siRNA therapy in the treatment of human pulmonary diseases (Lam et al., 2012). Christoph M. Zimmermann et al. developed a spray drying apparatus for the efficient delivery of lipid nanoparticle siRNA formulations. This method simultaneously preserves the structural integrity, cargo stability, and bioactivity, as well as gene-silencing efficiency of lipid nanoparticles (LNPs) (Zimmermann et al., 2022). Monica Agnoletti et al. employed a 3D-printed micromixer to fabricate siRNA-dendrimer nanocomplexes (Figures 10A–C), which were subsequently transformed into dry powder-based microparticles for inhalation via spray drying. This approach effectively preserves the structural integrity and biological activity of siRNA, facilitating the reconstitution of nanocomposites without compromising their functionality (Agnoletti et al., 2017). Drying powder formulation can also be employed for the pulmonary delivery of other biological macromolecules, including mRNA (Friis et al., 2023) and pDNA (Munir et al., 2022). Friis et al. developed a lipid nanoparticle-based mRNA formulation suitable for spray drying, demonstrating its ability to preserve drug stability and maintain mRNA functionality (Friis et al., 2023). Munir et al. employed nanospray drying technique to develop an inhalable dry powder formulation of RALA/pDNA nanoparticles for efficient gene therapy via pulmonary administration (Figure 10D) (Munir et al., 2022). The continuous pulmonary administration of siRNA and pDNA powder is a suitable approach for achieving more precise, rapid, and cost-effective assessment of the effects exerted by siRNA (Ito et al., 2019b).

The development of inhaled powder preparations requires the utilization of stable complex biological materials, along with different formulation strategies integrated into the manufacturing process. This ensures both biological and physical stability during production and throughout the warranty period (Chang et al., 2021). Dry powder preparations utilizing vectors (Keil et al., 2019; Jensen et al., 2012; Lam et al., 2012; Liang et al., 2014; Schulze et al., 2018; Mohri et al., 2010) such as chitosan (Mohri et al., 2010), PLGA (Jensen et al., 2012), and PEI (Keil et al., 2019) enable efficient pulmonary delivery of siRNA. The experiment conducted by Tobias W.M. Keila et al. demonstrated the feasibility of preparing nano-embedded particles containing nucleic acid for the production of suitable powder for inhalation (Keil et al., 2019). Mohri et al. also demonstrated the suitability of chitosan dry powder, prepared using the spray freeze-drying method, as a dosage form for lung gene therapy (Mohri et al., 2010). Furthermore, Schulze et al. developed a highly efficient spray drying technique to encapsulate polyethylenimine and lipid polymers within polyvinyl alcohol microparticles. This approach enhances nanoparticle retention time, mitigates cytotoxicity, augments transfection efficiency, and enables direct inhalation as dry powder (Schulze et al., 2018). Jensen et al. developed a cationic lipid-based modified PLGA nanoparticle powder formulation for targeted delivery of bioactive siRNA to the lung tissue (Jensen et al., 2012). However, the presence of a vector can have detrimental effects on drug efficacy, and several studies have demonstrated that utilizing naked DNA (without any delivery vector) can yield superior outcomes. Therefore, Xu et al. employed siRNA co-spray drying with mannitol and L-leucine to transform naked siRNA into an inhalable dry powder for the first time using the spray drying technique (Chow et al., 2017). Furthermore, Ito et al. demonstrated that the gene expression in mouse lungs was significantly higher when using pDNA powder composed of LHA compared to pDNA solution containing PEI and other bare pDNA powders, indicating that the composition of excipients also plays a crucial role in regulating gene expression in bare pDNA powder (Ito et al., 2019a). The preparation method of the dry powder has also been demonstrated to impact the formulation’s properties. For instance, vectors like mannitol are commonly used by Wanling Liang et al., and two methods, namely, spray drying (SD) and spray freeze drying (SFD), are employed for preparing pH-responsive peptides and plasmid DNA powders respectively. Both formulations exhibit favorable aerodynamic performance and biological activity; however, the spray drying powder demonstrates superior physical stability, higher transfection efficiency, and greater industrial potential (Liang et al., 2014).

Lung-targeted delivery of small interfering RNAs (siRNAs) holds promise for the treatment of viral respiratory infections, including influenza. Wanling Liang et al. developed inhalable spray-dried (SD) siRNA powder formulations incorporating pH-responsive peptides to facilitate efficient delivery of antiviral siRNAs against influenza (Liang et al., 2015). Dry powder inhalation therapy is widely employed in the treatment of both localized and metastatic lung cancer, presenting distinct advantages over injectable and nebulized aqueous formulations (Okamoto et al., 2011; Kuehl et al., 2020). Hirokazu Okamoto et al. formulated a chitosan mannitol powder incorporating two reporter genes, pCMV-Luc and pEGFP-F. The expression of the luciferase gene, driven by the CMV promoter (pCMV-Luc), and the plasmid DNA encoding acylated enhanced green fluorescent protein (pEGFP-F), exhibited elevated levels of gene expression in both normal and tumor tissues, as well as in intratracheal powders, in comparison to intravenous or intratracheal solutions (Figure 10E) (Okamoto et al., 2011). Philip J. Kuehl et al., conversely, devised a dry powder formulation for the inhalation administration of 5-aza, demonstrating enhanced stability in comparison to injection preparations. Moreover, aspirating powdered 5-aza exhibited improved pharmacokinetic characteristics in lung, liver, brain, and blood tissues, with the exception of lung tissues, when contrasted with nebulized aqueous preparations (Kuehl et al., 2020).

### 4.3 Pressurized metered dose formulations

Pressurized metered dose formulations (PMDIs) offer potential advantages over alternative pulmonary delivery systems, characterized by their portability, cost-effectiveness, rapid administration, and the capacity to store multiple doses in a compact tank. Metering valves ensure consistent dose delivery. As a result, PMDIs represent a more expedient alternative to nebulizers, particularly for treatments requiring frequent dosing (Bains et al., 2010). However, achieving particle dispersion stability in low dielectric HFA propellants poses a formidable challenge for PMDI (Conti et al., 2012). Incorporating the appropriate surfactant into the HFA matrix to enhance the fluidity of dry powders and mitigate particle aggregation by reducing surface tension and improving particle dispersion. This ensures uniform distribution of drug particles during inhalation. Denise S. Conti et al. employed a core-shell strategy to efficiently disperse cation-polymer-DNA nanoparticles in hydrofluoroalkane propellants, generating aerosols with exceptional characteristics from the corresponding PMDI. It was demonstrated that the propellant had no discernible impact on the biological activity of plasmid DNA (Conti et al., 2012). The key focus of the future development of pMDI system is still to solve the above problems. To sustain their position within the inhalation therapy market amidst rapid developments in DPIs and other SMIs, substantial improvements are indispensable (Smyth, 2005).

## 5 Summary and outlook

Transdermal and transpulmonary gene delivery aim to overcome challenges associated with systemic gene delivery, such as first-pass metabolism during oral administration, enzymatic degradation in circulation, and poor target specificity for systemic administration. The skin emerges as an ideal site for gene therapy due to its rich population of lymphocytes, keratinocytes, dendritic cells, and T cells. Transdermal gene delivery has shown potential in treating skin conditions such as epidermolysis bullosa (EB) and Netherton syndrome, as well as inhibiting skin inflammation by locally delivering siRNA for immunomodulation. However, transcutaneous gene delivery does present certain obstacles, such as limited cellular expression of genes or instability in gene transfer. Future advancements in transcutaneous gene delivery should prioritize optimizing equipment functionality, exploring delivery mechanisms, and ensuring technological safety.

Transpulmonary gene delivery not only allows for noninvasive drug administration but also mitigates drug degradation. The expansive alveolar surface area facilitates drug absorption and lowers the local required dosage, thereby minimizing potential side effects. However, the efficacy of inhaled gene therapy can be influenced by lung anatomy, physiological state, and metabolic characteristics. Increased mucus production and inflammation result in airway obstruction, hindering therapeutic agent delivery, necessitating specialized delivery systems for targeted pulmonary deposition. Considering the successful application of nucleic acid molecules in various lung disease treatments, it is crucial to develop inhalable and stable nucleic acid preparations for future clinical applications.

Interdisciplinary collaboration, technological innovation, and robust evaluation systems are necessary to overcome these challenges and achieve industrialization. Addressing these challenges is crucial not only for the advancement of gene therapy but also for realizing its substantial social value by providing patients with better therapeutic options.
